# Machine learning goes wild: Using data from captive individuals to infer wildlife behaviours

**DOI:** 10.1371/journal.pone.0227317

**Published:** 2020-05-05

**Authors:** Wanja Rast, Sophia Elisabeth Kimmig, Lisa Giese, Anne Berger

**Affiliations:** 1 Department of Evolutionary Ecology, Leibniz Institute for Zoo and Wildlife Research, Berlin, Germany; 2 Department of Ecological Dynamics, Leibniz Institute for Zoo and Wildlife Research, Berlin, Germany; University of Vermont, UNITED STATES

## Abstract

1. Remotely tracking distinct behaviours of animals using acceleration data and machine learning has been carried out successfully in several species in captive settings. In order to study the ecology of animals in natural habitats, such behaviour classification models need to be transferred to wild individuals. However, at present, the development of those models usually requires direct observation of the target animals. 2. The goal of this study was to infer the behaviour of wild, free-roaming animals from acceleration data by training behaviour classification models on captive individuals, without the necessity to observe their wild conspecifics. We further sought to develop methods to validate the credibility of the resulting behaviour extrapolations. 3. We trained two machine learning algorithms proposed by the literature, Random Forest (RF) and Support Vector Machine (SVM), on data from captive red foxes (*Vulpes vulpes*) and later applied them to data from wild foxes. We also tested a new advance for behaviour classification, by applying a moving window to an Artificial Neural Network (ANN). Finally, we investigated four strategies to validate our classification output. 4. While all three machine learning algorithms performed well under training conditions (Kappa values: RF (0.82), SVM (0.78), ANN (0.85)), the established methods, RF and SVM, failed in classifying distinct behaviours when transferred from captive to wild foxes. Behaviour classification with the ANN and a moving window, in contrast, inferred distinct behaviours and showed consistent results for most individuals. 5. Our approach is a substantial improvement over the methods previously proposed in the literature as it generated plausible results for wild fox behaviour. We were able to infer the behaviour of wild animals that have never been observed in the wild and to further illustrate the credibility of the output. This framework is not restricted to foxes but can be applied to infer the behaviour of many other species and thus empowers new advances in behavioural ecology.

## Introduction

Animal-borne sensors such as temperature loggers, salinity loggers or microphones are used to study a wide variety of parameters in wild animals without disturbance by human observers [1]. In the study of movement ecology of species [2], animal-borne sensors make it possible to track the locations of wild animals. The first attempts to remotely track animal locations were made in the 1960s through VHF telemetry [3]. In more recent years it has become common practice to track animal locations with satellite systems [4], enabling researchers to study where individuals dwell. However, the spectrum of ecological questions that can be addressed by using location data alone is limited. By combining such data with behavioural data, more in-depth studies of species will become possible [5]. Yet, in contrast to recording locations, remotely tracking the behaviour of free-ranging animals is not well established at this point.

The principal underlying remote-tracking of behaviour is to attach accelerometers to animals to record their body movement. The first major study utilizing acceleration data to study the behaviour of animals was conducted in 1996 [6]. Since then, many studies have shown that acceleration data can be used to infer the behaviour of animals by employing various machine learning algorithms [5, 7]. To train these algorithms for pattern recognition and data classification, the acquisition of acceleration data was coupled with direct observation of the behaviours of the tagged animals. Using one portion of this ground-truthed data set to train the algorithm and another portion to infer behaviour from it allows validation of the inferred behaviour.

Extrapolating behaviours from acceleration data of wild individuals is a challenge since it is often impossible to test whether the extrapolated behaviours are correct or not. Some models were trained and validated on the same wild individuals [8–10], which requires direct observation of the studied individuals at least for a certain period of time. However, the promising advance of behaviour classification through machine learning is the ability to study the behaviour of wild animals without observing (and possibly disturbing) them. Furthermore, direct observation may often not be a feasible option, especially when target species are elusive or cryptic.

For other models, additional sensors such as GPS [11] or depth and speed sensors for aquatic species [9, 10, 12] were employed to identify the behaviours executed. In these cases, the information from the additional sensors was used to investigate the behavioural context the animal was in at the time of data recording, in order to delimit likely behaviours. For studies in which no validation was possible, various behaviours were grouped into broad, easily distinguishable categories to reduce confusion of similar behaviours [13, 14]. Thus, accurately inferring distinct behaviours of wild individuals still poses a problem.

The Random Forest (RF) and the Support Vector Machine (SVM) are popular approaches to infer animal behaviour from acceleration data and have yielded good results under training conditions [5, 15]. Yet, to our knowledge, there are no studies successfully transferring a behaviour classification model trained on captive individuals to wild individuals.

To study the complex behaviour- or movement ecology of wild animals, however, a valid data set of linked GPS locations and behavioural data is needed. In this study, we, therefore, aim to test the capacity of different machine learning algorithms in inferring the behaviour of wild foxes (*Vulpes vulpes*) from acceleration data. We provide a framework to infer the behaviour of wild red foxes based on an Artificial Neural Network (ANN) trained on captive red foxes.

Our framework further addresses the issues of working with small training data sets (a common obstacle in wildlife- and conservation research) by using a new approach to efficiently exploit the given data set. Finally, we suggest how to validate the inferred behaviours when observation of free-ranging individuals is not a feasible option. We propose four strategies to assess the credibility of the output by combining the classified behaviour with GPS and temporal information. The study set-up, together with our novel approach, enables us to test the use of machine learning for behaviour classification and to empower behaviour classification of wildlife through acceleration data in the future.

## Material and methods

### Data collection and acceleration logger setup

Animal catching and handling have been approved from the State Office for Health and Social Affairs, department of veterinary affairs (permit number: IC113-G0211/15) and the ethics committee of the Leibniz Institute for Zoo and Wildlife Research in Berlin (permit number: 2015-03-04) and have been conducted according to applicable national and international guidelines. Approvals have been received prior to beginning research. To reduce stress during handling, all foxes got anesthetized before the deployment of radio collars. For anaesthesia we first used a long established mixture of Xylazin (10-16mg/kg) and Ketamin (12-20mg/kg) and later switched to an improved mixture of Ketamin (4mg/kg), Medetomidin (70μg/kg) and Midazolam (0,6mg/kg) that is better tolerated.

**Fig 1:**
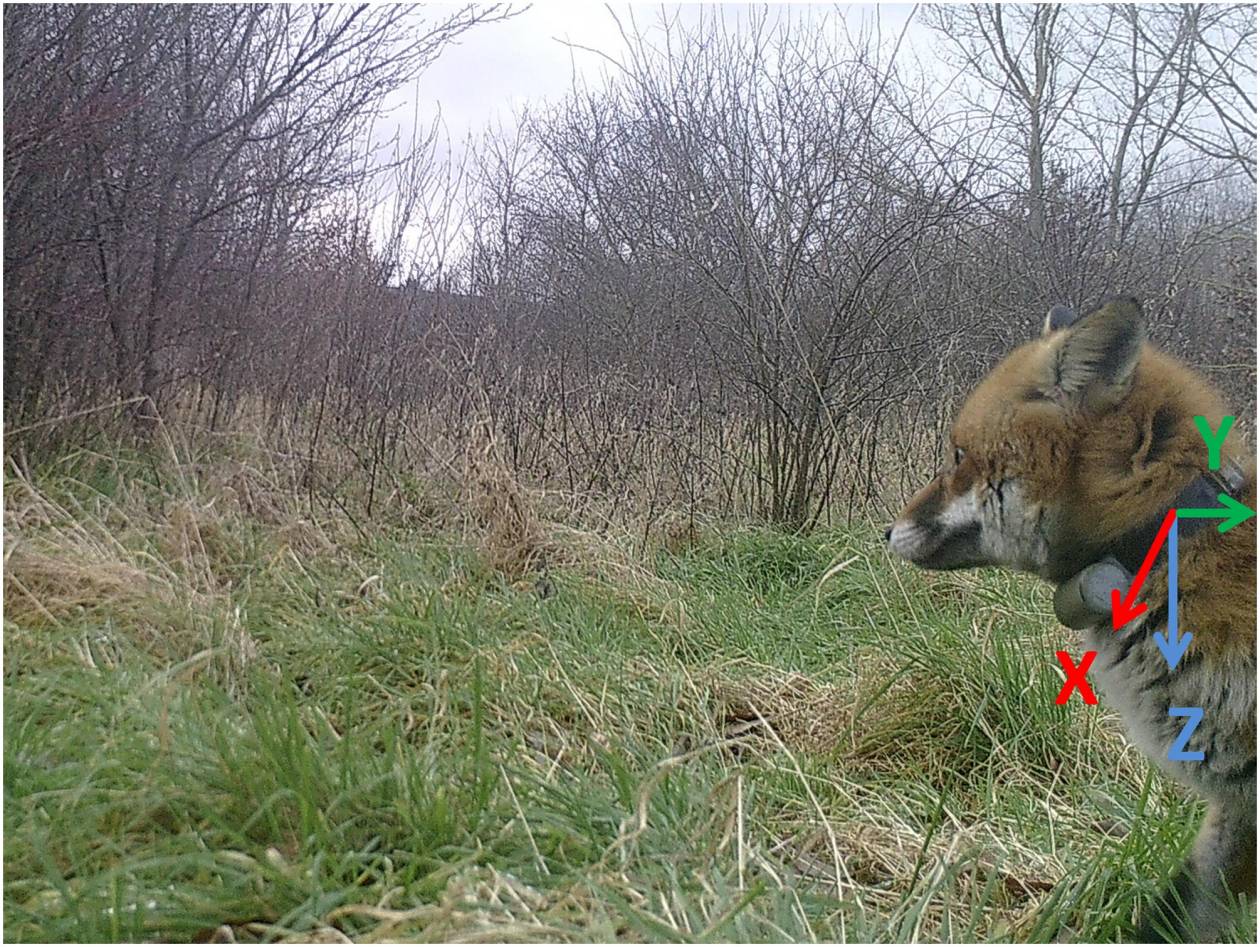
Camera trap picture of a wild red fox (“Gerlinde”), collared in Berlin in 2016. The arrows symbolize the X-, Y- and Z-axis (corresponding to sway-, surge- and heave-motion).

For gathering the acceleration and GPS data sets, used in this study, we deployed UHF-GPS collars (“1C-light” and “1C-heavy”, E-obs GmbH, Munich, Germany; Fig 1) on adult red foxes, both, in captivity and in the wild. Both captive and wild individuals were tagged with the same type of sensors and acceleration data logger settings. Captive individuals were observed to train and test the models and wild individuals were used to apply them. For the training data set, two individuals (female, approx. 8 years old) were collared in a game park enclosure in the north-west of Berlin between November 2015 and June 2016. Their enclosure mainly consisted of a sandy and stony substrate and was partially covered with concrete, grass and weeds. Several trees, tree roots, piles of stones and cement tubes provided a heterogeneous environment with both opportunities to hide and climb. One of the cement tubes led to an artificial, observable den. The two foxes were chosen to be collared, because of their lacking fear towards visitors and noises and the resulting possibility to be observed outside their den for several hours per day. For the field dataset, data from wild foxes were used that were radio-collared by Kimmig et al. in the city of Berlin, Germany, between 2015 and 2018. In total 17 wild individuals (10 female, 7 male) were caught. Out of those, for 9 individuals (7 females, 2 males), three consecutive months of data were available and they were therefore included in the analysis. All individuals were adults (with ages ranging from 1.5 to 7 years) and their urban and suburban habitats were characterized by a heterogeneous structure, including green spaces as well as concrete.

The acceleration loggers that were embedded in the UHF-GPS collars were set up to measure acceleration in short intervals at a frequency of two minutes. Data was recorded for three axes perpendicular to each other at a sampling rate of 33.33Hz per axis. There were 110 acceleration measurements taken for each axis in each measurement interval. Resulting from the sampling rate and the number of measurements for each axis the duration of each recording interval was 3.3 seconds. We refer to a single recording interval as a burst.

To train the algorithms we used the raw ground-truthed data of the captive foxes that were observed during the recording of acceleration data. A specific UHF-pinger signal indicated the start of each burst for the observer who then noted the displayed behaviour. All measured behaviours had been previously classified in an ethogram that was established through observations before and after collaring the individuals (with all steps conducted by the same observer). It contained the following behaviours: feeding, grooming, resting, caching, trotting and walking (for a detailed description of the behaviours see S1 Table). The pinger signal could be detected acoustically with a UHF Wide Range Receiver that was set to the unique frequency of the collars (see [16]) and was not audible to the foxes.

During a burst, the animal in focus was observed closely and the behaviour was noted. Each observation was linked to the corresponding acceleration burst via the unique timestamp.

Due to slight shifts in the collars’ timestamps, the raw acceleration data of a number of consecutive bursts—ideally encompassing a distinctive change in behaviour (e.g. resting followed by trotting)—was visually inspected and compared to the noted behaviours. The timestamps of observations were corrected accordingly.

After excluding all bursts containing more than one behaviour, 4159 bursts of six different behaviour classes were used as the input for the model training (feeding: 367, grooming: 1140, resting: 2114, caching (bury food to consume it later): 197, trotting: 179, walking: 162).

### Data preparation

We calculated summary statistics from the raw acceleration data, separately for each burst, to serve as predictors for the machine learning algorithms. The following predictors were calculated per axis: mean, standard deviation, inverse coefficient of variation, variance, skewness and kurtosis. Additional predictors represent combinations of all three axes and were calculated according to the corresponding literature: q [5], pitch and roll [17] and overall dynamic body acceleration (ODBA) [18]. In addition to the summary statistics, we added the whole spectrum of a Fast Fourier Transformation of each axis to the set of predictors. As most of the time-related information in the raw acceleration data is lost when calculating the summary statistics we decided to use the full spectrum to utilize this information. For a complete list of predictors see S2 Table. We performed all data transformations and the construction of the ANN in R [19] and Rstudio [20]. The sum_data function in the accelerateR package (W. Rast, unpublished data.) was used for summary statistics and Fast Fourier Transformation calculation.

### Data classification

#### Established methods: Support vector machines (SVM) and random forest (RF)

Support Vector Machines separate data of different classes from each other by constructing a hyperplane between them. Classification of new data is subsequently based on their relative position to the hyperplane. By default, the classification is binary. For applications with multiple classes, more hyper-planes between classes will be constructed [21]. We used the implementation of an SVM in the R package “e1071” [22] with the kernel type “radial”.

Random Forests are an improvement of the classical Classification and Regression Trees (CART) [23]. While in CART all predictors are used, the RF picks a random subset of predictors to fit a tree. This is repeated several times, and the final prediction is the result of all trees combined by a majority rule [24]. We used the implementation of an RF in the R package “randomForest” [25] with the standard settings using 500 trees.

#### Artificial neural network (ANN)

ANNs are similar to biological neural networks and consist of multiple nodes that are distributed over several layers and interconnected [26]. Nodes are activated based on the input variables (predictors) and an activation function. In the simplest cases, this function is a summation of all input variables that are passed to a specific node. These functions also include weights that change the influence of every input variable and are set during the training phase. For training, a ground-truthed data set is needed on which the ANN establishes the node connections and the weights so that the output of the ANN corresponds to the target classes of the model data. The activation or non-activation of nodes serve as input for the next layer of nodes. The last layer usually consists of nodes representing the target classes. Their activation leads to the assignment of data to a class.

For our study, we chose a three-layer network with the output of the last layer being a specific behaviour class. We used a feed-forward type architecture for the ANN and used the Keras package [27] to implement it.

### Moving window

One strategy that has been tested with continuously recorded data is to apply a moving window to partition the acceleration data and to compute summary statistics for each of the resulting segments. In different studies, these windows could partially overlap or not overlap at all [28–30]. An application example very similar to our approach is the assessment of car driver aggressiveness using continuous data by Ferreira et al. [31]. However, to our knowledge, this approach has never been used on burst data in wildlife ecology.

**Fig 2:**
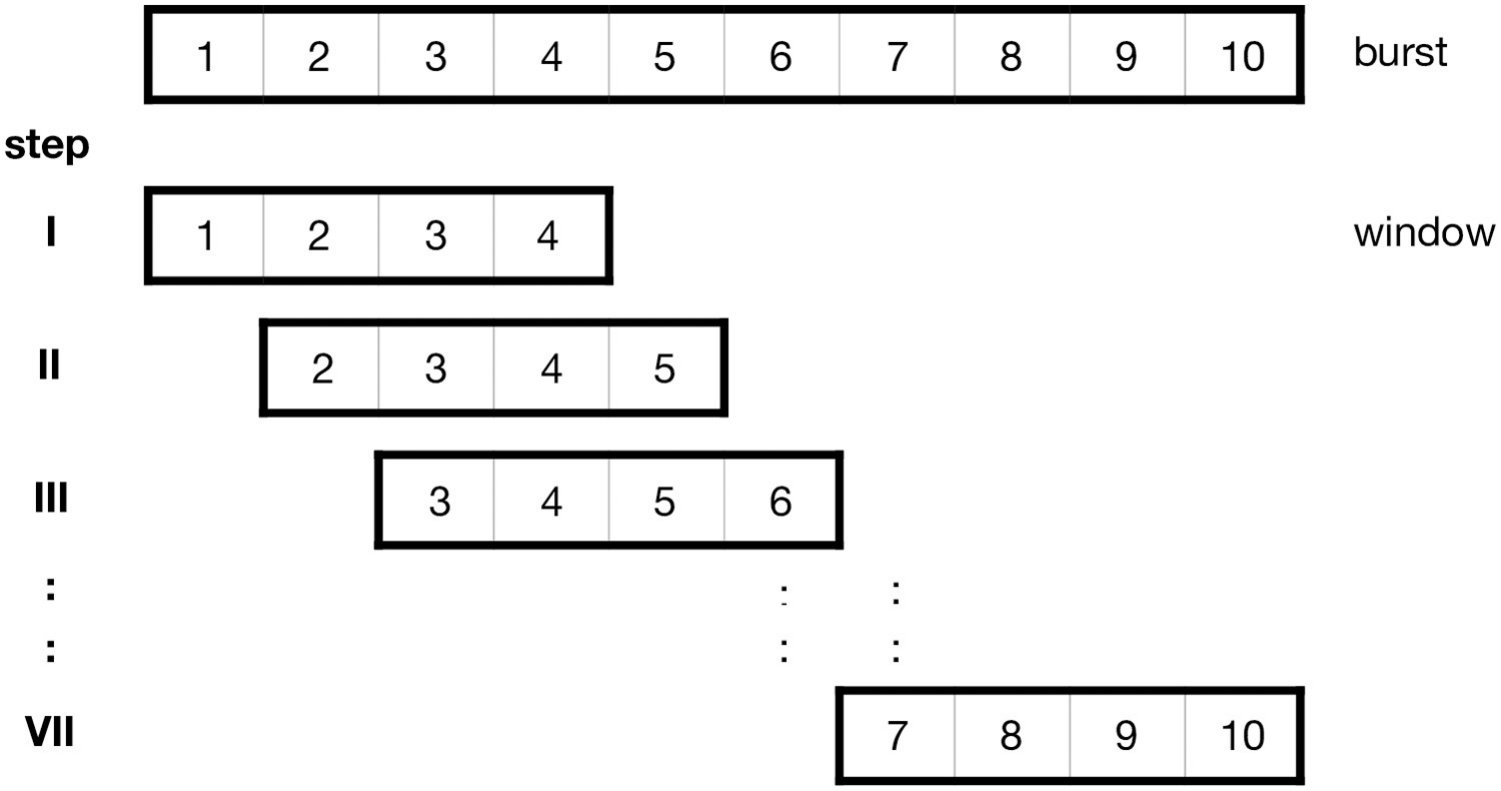
Schematic representation of the moving window approach: Starting at the beginning of a data set (“burst”, here n = 10), a fixed number of consecutive data (“window”, here n = 4) is taken out and analysed. In the further step-by-step analysis, the window is shifted by one data set until the window has reached the end of the complete data set (7 steps in the schematic example).

We applied a moving window to every recorded burst to increase the sample size of our data set since it was found that ANNs show better performance with increasing sample size [32, 33] and require large training data sets [34]. In the first set, this window reduced the amount of data within the burst from the original 110 measurements down to a subset of the window length. We then computed the summary statistics and Fast Fourier Transformation (S2 Table) for this subset. In a second step, the window was moved by one position so that the first measurement of every axis was removed and one new measurement for every axis was added to the end of the window (see Fig 2). We then computed all variables for the second window and so forth. The window was moved until it included the last measurement of the original burst, resulting in a number of predictor sets representing the same burst. In contrast to extracting random subsets, this approach preserves the order of the data for a specific behaviour, and we were able to calculate the Fourier spectrum which is dependent on the correct order of measurements.

### Model evaluation

To evaluate the model performance of all three machine learning algorithms, we trained them on 70% of the data (training data). We then inferred the behaviour for the remaining 30% (test data) by classifying them with the trained model and assigning a specific behaviour to each burst accordingly (or assigning “other”, respectively as described below). Since the number of observations per behaviour class differed, we split the data of each behaviour class separately in a random fashion so that the original proportions of behaviour counts were similar in the training and test data sets. We applied the moving window to the training and test sets after the split. We calculated the recall (true positives / (true positives + false negatives)) and precision (true positives / (true positives + false positives)) [7] for each behaviour. For the sake of completeness, we also calculated the accuracy ((true positives + true negatives) / total number of samples). For comparison with other models we calculated Cohens Kappa with *Kappa* = (*p*_*o*−_*p*_*e*_)/(1 − *p*_*e*_) with $p_{o}=\sum_{1}^{c}{(TP}_{c}/n)$ and $p_{e}=\sum_{1}^{c}{((TP}_{c}+{TN}_{c})/n*({TP}_{c}+{FP}_{c})/n)$. With <n> being the total sample size and <c> the number of classes [35].

To reduce confusion of behaviours, a threshold was set for the ANN assignments. Only behaviour assignments that exceeded a probability of 0.7 were accepted. All assignments below that threshold were classified as “other” behaviour. This was necessary to account for the fact that captive individuals may not execute the full range of behaviours available to the species, which would lead to some behaviour (e.g. hunting or fighting) not being included in the model. If wild individuals displayed any of these behaviours, they could be incorrectly classified as one of the behaviours included in the model. We expect that such classifications would be assigned at low probability so that we can avoid these errors by implementing the threshold. Similarly, recordings in which the individual changed its behaviour during a burst should not be characteristic for any specific behaviour and therefore should also fall below the threshold.

### Model selection

Artificial Neural Networks are used for a variety of tasks such as image recognition, sentiment analysis or regression. The necessary sample size and ANN architecture depend on the specific task [36]. Finding the optimal properties for the best performing ANN is not achieved by a scientific method but rather by trial and error [33]. To find the best window size we trained the ANN on window sizes from 20 to the full 110 and finally decided on 79. We evaluated all models by calculating the recall, precision and the proportion of “other” behaviours. As recall and precision are calculated for each behaviour, we first computed their means and then calculated the mean of the resulting mean recall and precision as well as the proportion of “other” behaviours. The latter was subtracted from one to be on the same scale as recall and precision. A General Additive Model (GAM) was applied to the calculated means for all window sizes. We calculated the slope m of the GAM fit for each window size using the difference quotient m = (Δy_n_-Δy_n-1_)/(Δx_n_-Δx_n-1_). Variable x corresponds to the window size and y to the calculated model performance, n corresponds to a specific window size and n-1 to the previous window size. A window size of 79 provided the best trade-off between small window size and high performance (see “Model selection” in the Results).

### Application to wild individuals

For our subsequent analysis of behaviour inference, we selected wild foxes for which at least three consecutive months of acceleration data were available (N = 9). We considered all months in which data was recorded for at least half of the month. In addition to the acceleration data, the tags recorded GPS locations every four minutes for the first eight weeks, after that every 20 minutes (GPS for fox “Gerlinde” was only recorded every 20 minutes). Using acceleration informed GPS measurement, this interval was reduced to every four hours when a fox was inactive. We trained all three classification models on the complete ground-truthed dataset of the captive foxes and applied the trained model to classify the data of the wild foxes. As the moving window results in multiple behaviour outputs for each burst, only one behaviour was assigned to each burst, following majority rule. We consider all classifications within one burst as equal and determine the absolute majority.

### Validation of behaviour assignments

We assessed the plausibility of the ANNs’ behavioural assignments by examining the following four aspects: (i) biological credibility of the behaviour assignments (ii) consistency over individuals and time (iii) coherence with the GPS data and (iv) coherence with ODBA.

To address biological credibility (i), we calculated the time-dependent composition of behaviours throughout the day and compared it to the literature on fox behaviour. As seasonal shifts can influence behavioural compositions, we separated the behaviour assignments by month. For each day within a single month, we counted the number of assignments of each behaviour (for each minute covered by the tag schedule) in the 24 hours. We further used the corresponding plots to (ii) visually compare the daily patterns over time and between individuals. (iii) We incorporated the given GPS information of the free-ranging individuals because we expected the GPS data to correspond with specific behavioural classes. For instance, spatial clustering of GPS data should correspond with stationary resting behaviour. We treated points as a cluster when consecutive GPS points were within a 50m radius of the first GPS point of that cluster. Points recorded more than 50m away were defined as the first point of a new cluster. Since it was possible that clusters consisted of only a single point, we only considered behaviour assignments to be spatially clustered when at least 10 classified behaviour items were assigned to the same cluster. We then calculated for each behaviour the proportion of behaviour assignments that were within a cluster. In addition, we investigated the coherence of GPS based speed measure and movement-related behaviour classifications (trotting and walking). We, therefore, calculated the speed of the moving animal based on the spatial and temporal distances between consecutive GPS points. Due to independent schedules, GPS and acceleration data were not recorded exactly simultaneously. Hence acceleration data that was recorded within 10 seconds of a GPS measurement were considered. Finally, we (iv) compared the temporal distribution of ODBA values and behaviour assignments by constructing actograms using accelerateR.

## Results

### Training conditions: Captive foxes

We could classify all six behaviours during the validation using SVM and RF. Classification success differed between the behaviour classes for both algorithms. We achieved the best classification success for resting and the lowest for caching and walking (Table 1). The confusion matrices (S3 and S4 Tables) showed that grooming and walking were confused more often compared to other behaviours. Recall can be interpreted as the proportion of behaviour events that were correctly classified. Feeding (SVM), for example, had a recall of 0.43, meaning that 43% of all feeding events were correctly classified as feeding. Precision can be interpreted as the probability for an assignment to be correct. Feeding had a precision of 0.58, meaning that a single assignment of feeding is correct with a chance of 58%. Both algorithms show comparable results only for resting. The SVM performs worse for all other behaviours. Our initial testing showed that the SVM performed better without the addition of the FFT spectrum but we kept the model this way to ensure the comparability of all three models. We added the accuracy metric that is often used for model evaluation but will not endorse its use for this study: As accuracy uses the true negatives it is influenced by the large number of resting observations that we got. Since most of the resting data is classified correctly these data is treated as true negatives for all other behaviour classes and thus resulting in higher accuracy values for those behaviour classes.

**Table 1:** Recall and precision of the classification output compared for support vector machine (SVM), random forest (RF) and artificial neural network (ANN). All algorithms are capable of classifying and inferring fox behaviour with a high success rate (exceptions are caching and walking for SVM and RF).

	Feeding	Grooming	Resting	Caching	Trotting	Walking
**SVM**						
recall	0.43	0.33	0.98	0.37	1.00	0.27
precision	0.58	0.70	0.98	0.21	0.19	0.36
accuracy	0.92	0.77	0.94	0.90	0.81	0.95
**RF**						
recall	0.70	0.93	0.92	0.68	0.96	0.43
precision	0.71	0.80	0.99	0.59	0.91	0.84
accuracy	0.94	0.91	0.95	0.96	0.99	0.98
**ANN**						
recall	0.83	0.88	0.96	0.67	0.96	0.74
precision	0.84	0.95	0.98	0.83	0.91	0.71
accuracy	0.93	0.92	0.95	0.95	0.96	0.95

The Kappa values for the RF and SVM are 0.81 and 0.51 respectively. Our initial testing with training both models without the Fast Fourier Spectrum resulted in Kappa values of 0.81 and 0.78 for RF and SVM respectively. The performance of the RF remains the same while the performance of the SVM decreased due to the addition of the Fast Fourier spectrum.

Like RF and SVM, the ANN could predict all six behaviours during validation. Also, classification success differed between behaviour classes. The performance of the ANN is overall comparable to the RF but performs better than the SVM. The confusion of walking behaviour with grooming is reduced compared to the SVM and RF (S6 Table). The kappa value of 0.85 for the ANN was also higher than for the RF (0.81) and SVM (0.51). The proportion of assignments that did not surpass the threshold was 0.04.

**Fig 3:**
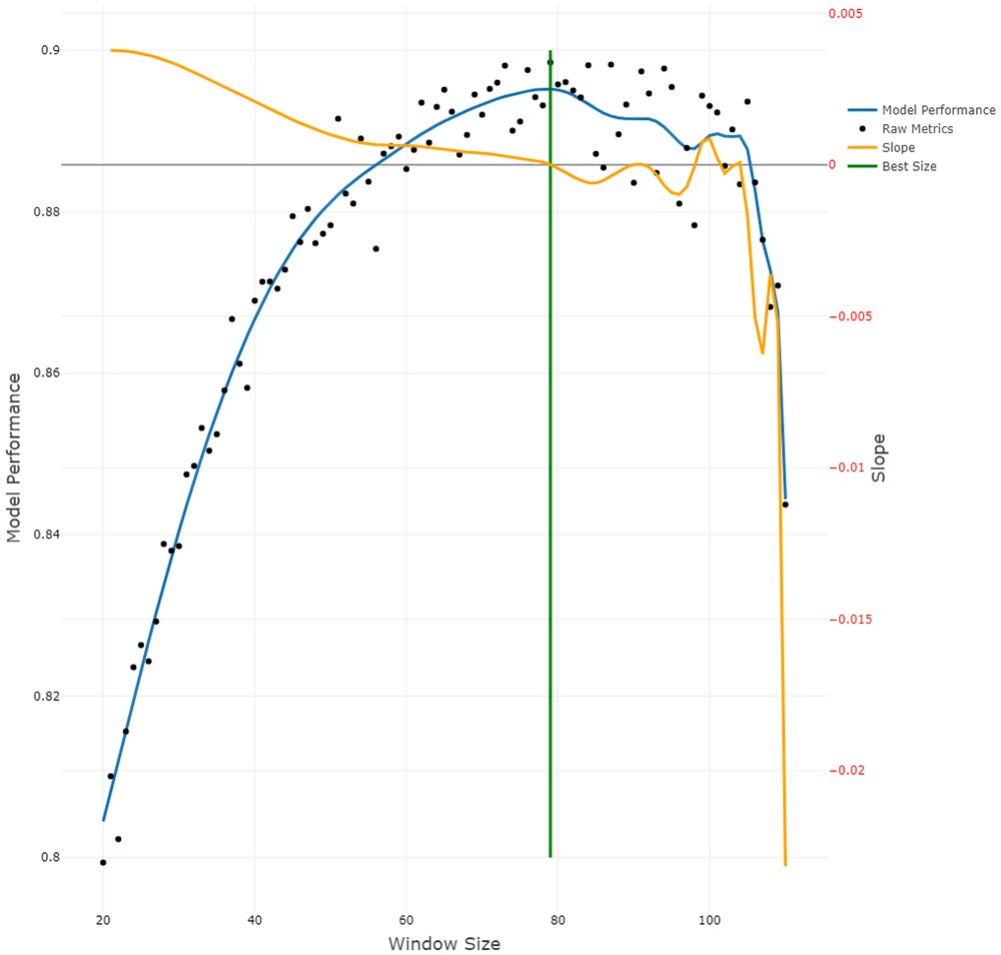
ANN model performance in relation to window size. Black dots show the computed performance values. The blue line is the result of a General Additive Model, k = 40 [37] fit. The y-axis on the left side labelled “Model Performance” corresponds to the Model Performance line (blue) and Raw Metrics points (black). The orange line is the calculated slope of the model performance, which corresponds to the y-axis on the right side labelled “Slope”. The green vertical line represents the best window size of 79.

Model performance of the ANN appears to be dependent on the window size (Fig 3) and decreases towards both ends of the window size spectrum. Smaller window sizes seem to have a stronger impact on model performance than larger window sizes. The GAM fit has its maximum at window size 79, with the slope of the GAM fit close to 0. We thus considered 79 to be the best trade-off between model performance and window size and used it for the final model (see Discussion).

### Field conditions: Application to wild foxes

We here show the results for all wild foxes and plots for those two wild foxes (“Que” and “Gerlinde”), whose collars yielded data over a whole year. Graphic representation of all remaining individuals is presented in the supplemental material (S1–S7 Figs).

When the trained SVM and RF models were applied to classify the behaviour of the nine wild foxes, all bursts were classified as grooming. No resting, caching, feeding, trotting or walking events were detected (Table 2). When applying the trained ANN to the wild fox data, all six behaviour categories were assigned in all nine individuals (Table 3). For the field data of “Que” and” Gerlinde”, a proportion of 1% did not exceed the 70% threshold and was therefore labelled “other”. For both foxes feeding, caching and walking were assigned at low rates.

**Table 2:** Number of occurrences of every classified behaviour for the wild foxes. Count of all behaviour assignments compared for support vector machine (SVM) and random forest (RF). Overall, all foxes show similar proportions of behaviours throughout their measurement periods. As all individuals were tagged for different time periods, the absolute number of assignments differs between individuals.

Individual	Measure	feeding	grooming	resting	caching	trotting	walking	other
**SVM**								
Gerlinde	count	0	289248	0	0	0	0	0
Gisel	count	0	102920	0	0	0	0	0
Hazel	count	0	103951	0	0	0	0	0
Ida	count	0	110337	0	0	0	0	0
Jack	count	0	243742	0	0	0	0	0
Kyna	count	0	159211	0	0	0	0	0
Nikita	count	0	72653	0	0	0	0	0
Porthos	count	0	149254	0	0	0	0	0
Que	count	0	274792	0	0	0	0	0
**RF**								
Gerlinde	count	0	289248	0	0	0	0	0
Gisel	count	0	102920	0	0	0	0	0
Hazel	count	0	103951	0	0	0	0	0
Ida	count	0	110337	0	0	0	0	0
Jack	count	0	243742	0	0	0	0	0
Kyna	count	0	159211	0	0	0	0	0
Nikita	count	0	72653	0	0	0	0	0
Porthos	count	0	149254	0	0	0	0	0
Que	count	0	274792	0	0	0	0	0

**Table 3:** Number of occurrences of every classified behaviour for the wild foxes. Count and proportion of all behaviour assignments of the artificial neural network (ANN). Overall, all foxes show similar proportions of behaviours throughout their measurement periods. As all individuals were tagged for different time periods, the absolute number of assignments differs between individuals.

Individual	Measure	Feeding	Grooming	Resting	Caching	Trotting	Walking	Other
Gerlinde	count	2288	78194	171890	1016	30020	1664	4176
	proportion	0.005	0.23	0.61	0.007	0.12	0.02	0.01
Gisel	count	3019	16545	61405	1464	10766	7193	2528
	proportion	0.03	0.16	0.60	0.01	0.10	0.07	0.02
Hazel	count	2887	26014	57094	1319	8570	4874	3193
	proportion	0.03	0.25	0.55	0.01	0.08	0.04	0.03
Ida	count	1311	25162	65354	241	12848	1612	3789
	proportion	0.01	0.23	0.59	0.002	0.12	0.01	0.03
Jack	count	3420	136086	72507	523	17116	5092	8996
	proportion	0.01	0.56	0.30	0.002	0.07	0.02	0.04
Kyna	count	1070	45185	104491	266	6016	750	1433
	proportion	0.007	0.28	0.66	0.001	0.04	0.005	0.009
Nikita	count	2318	10430	47996	465	2678	7639	1127
	proportion	0.03	0.14	0.66	0.006	0.04	0.11	0.02
Porthos	count	1266	32624	91370	3555	16236	530	3673
	proportion	0.008	0.22	0.61	0.02	0.11	0.004	0.02
Que	count	1317	64092	166349	1875	32531	5749	2879
	proportion	0.008	0.27	0.59	0.004	0.10	0.006	0.01

### Validation and credibility of the behaviour assignments

#### Biological credibility of the behaviour assignments (i) and consistency over individuals and time (ii)

**Fig 4:**
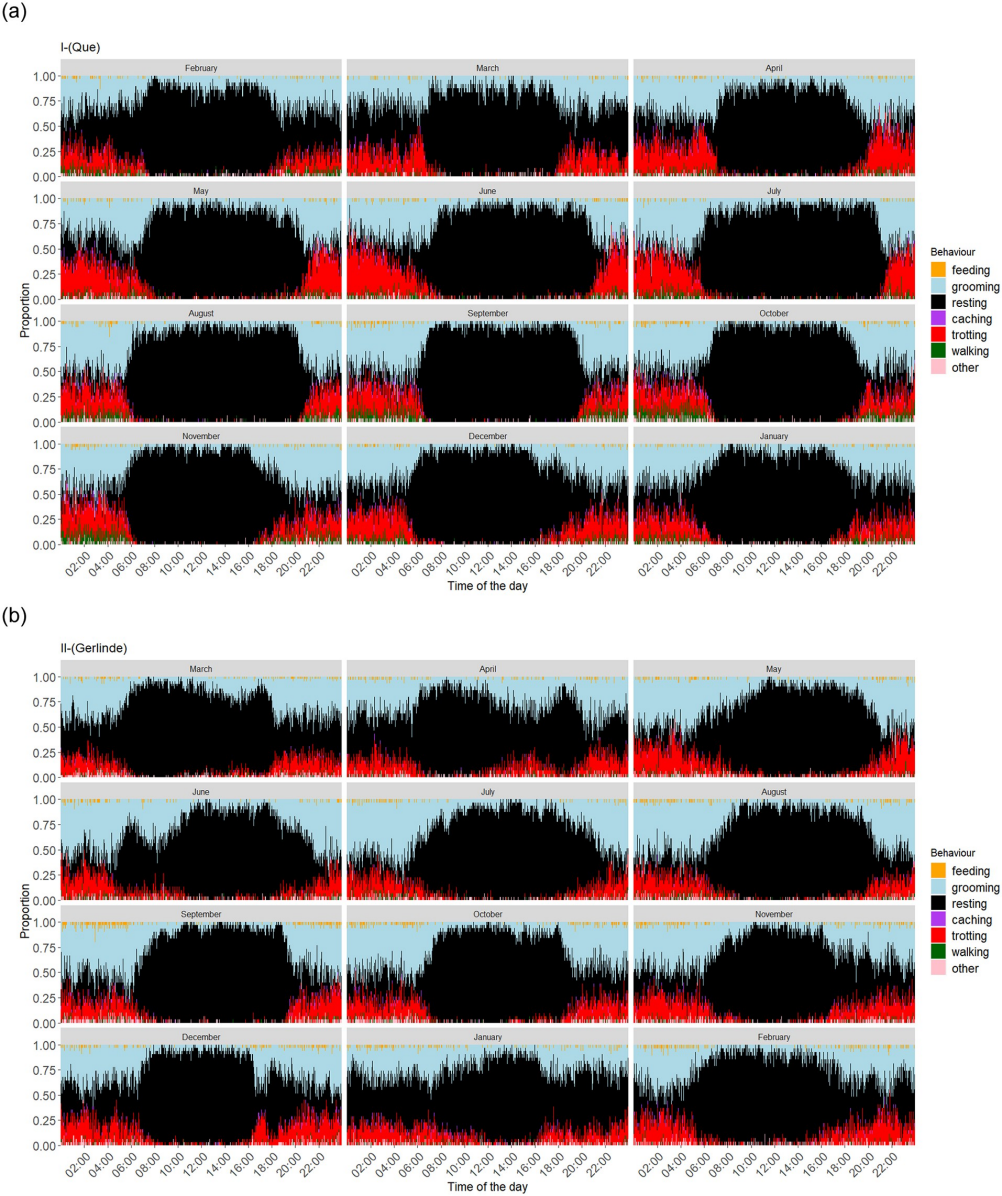
Time-dependent composition of behaviours of Que (I) and Gerlinde (II). Stacked bars represent the proportion of each behaviour at a given time of day, in each month. The data showed here span from February 2018 to January 2019 for Que and from March 2016 to February 2017 for Gerlinde.

Looking at the time-dependent composition of each individual’s behaviour (Fig 4, S1–S7 Figs), a similar pattern of behavioural composition over time is clearly noticeable (months without full data recording ought to be excluded for feasible interpretation). Clearly, there is a high proportion of resting behaviour during the middle of the day, while trotting is mostly inferred during dark hours. Trotting is also inferred more often than walking. There seems to be a seasonal change in resting behaviour, with resting events being more explicitly limited to the daytime in summer months. Feeding events are more often inferred during dark hours than during the daytime, when mostly resting and some grooming are classified.

In the comparison between individuals some differences emerge. Some individuals, e.g., show less trotting (S2 Fig), more walking (S3 Fig) or much more grooming than others (S7 Fig). Despite this variation, the general pattern of behaviour composition appears very similar across all individuals.

#### (iii) Coherence with GPS

**Fig 5:**
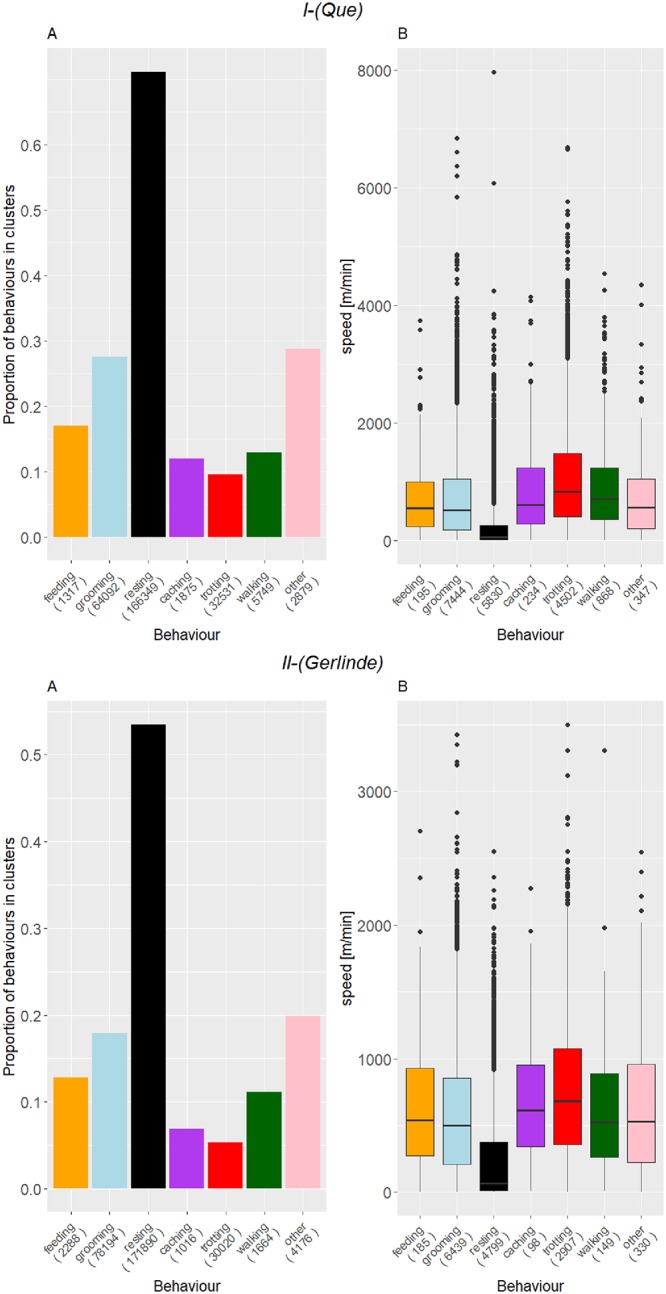
Behaviour assignments of Que (I) and Gerlinde (II) in relation to GPS clusters (A) and speed (B). The numbers in the brackets indicate the sample size of each behaviour class. (I) Resting shows the highest association with GPS clusters (71%) and trotting the lowest (9%). Resting events are associated with significantly lower speed than trotting events (Wilcoxon rank sum test, W = 3024826, p < 0.001). (II) Resting shows the highest association with GPS clusters (53%) and trotting the lowest (5%). Resting events are associated with significantly lower speed than trotting events (Wilcoxon rank sum test, W = 2286090, p < 0.001).

Resting behaviour appears to be highly associated with GPS clusters (Fig 5A), while all other behaviours are inferred mostly outside of clusters. This also applies to all other wild foxes (S8A–S14A Figs). For the analysis of the correspondence between behaviours and GPS based speed measurements, we used only acceleration data recorded within 10 seconds of a GPS fix (for Que: 7%; Gerlinde: 5%). Resting events were classified at lower GPS-based speed than trotting events (Fig 5B, S8B–S14B Figs).

#### (iv) Coherence with ODBA

**Fig 6:**
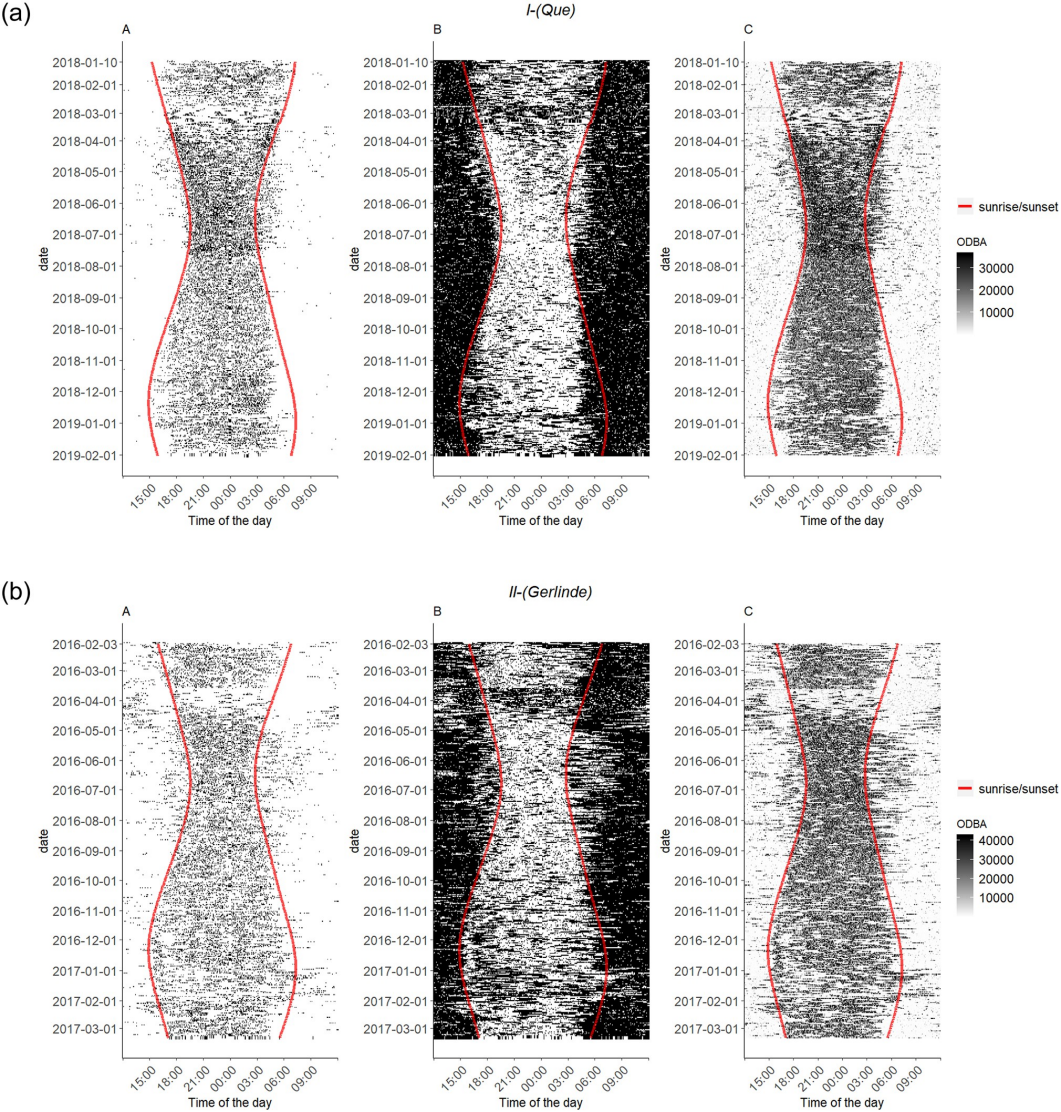
Temporal distribution of trotting (A), resting (B) and ODBA values (C) for Que (I) and Gerlinde (II). The red lines indicate sunset and sunrise. (A) Black spaces indicate times at which trotting behaviour was classified, whereas white spaces indicate the classification of all other behaviours. (B) Black spaces indicate times at which resting behaviour was classified, whereas white spaces indicate classifications of all other behaviours. (C) Higher ODBA values are indicated by darker spaces.

The actograms show that trotting is predominantly classified at times when ODBA values are high. Trotting, as well as high ODBA, occur mostly during night-time. Resting, in turn, is most often classified at times with low ODBA values (Fig 6). This is also valid for all remaining foxes (S15–S21 Figs).

## Discussion

In the present study we sought to advance the abilities to remotely assess the behaviour of animals in the wild without directly observing (and respectively disturbing) the target animals. We therefore, tested the capacity of three machine learning algorithms (SVM, RF and ANN) to infer wild fox behaviour after training with a ground-truthed data set of two captive red foxes. The performances of the RF and the ANN were on similar levels under training conditions, the SVM overall performed worse than the other two. The ANN with the moving window approach, however, was able to infer caching and walking behaviour much better than the other two. Both RF and SVM generally performed well in inferring behaviour during validation (Table 1) and showed comparable results to other studies [5, 38, 39]. When applied to the wild foxes, however, they both failed to discriminate the different behaviours (Table 2).

The application of a model trained on one individual to a conspecific (that the model was not trained on), is crucial to bring this method into practical use, yet this remains a major obstacle. One rare example for a study classifying behaviours of conspecifics is the study conducted by Moreau et al. [40] who used 3D-accelerometers on three goats to determine their head position (at accuracies of 61–82%) but could only predict three different traits, showing that inter-individual model projection leads to a reduction in the prediction’s accuracy.

### Support Vector Machine (SVM) and random forest (RF)

When applied to the data from wild foxes, SVM and RF classified all behaviours as grooming. Considering that these datasets contain measurements for at least three months and that the GPS signal clearly showed the animals covering large distances, this classification is clearly unrealistic. In all cases, the models were trained and validated on measurements from the same captive individuals. Since both models correctly inferred behaviours of those captive individuals during validation, we suggest that SVM and RF thus failed to recognize similar patterns between wild and zoo-kept individuals because wild foxes showed characteristics in their behaviour that were too different from the captive foxes to be detected by those approaches.

This could be solved by training the model on ground-truthed data obtained from observing wild individuals that are logged [13]. However, as mentioned above, this is not always feasible, so it would be highly desirable to do trait classification without the necessity to directly observe wild animals. An additional problem of SVM and RF could be the recording of mixed behaviours. During model training, all recordings with mixed behaviours within one burst were excluded from the data set. However, it is a fair assumption that at least some recordings from wild individuals do contain more than one behaviour. Even though we implemented a probability threshold to account for these mixed bursts and—in the best case—classify them as "other" behaviour, these recordings might still pose an unclassifiable problem. Another issue is that grooming is classified to such a high proportion by both SVM and RF. As grooming behaviour is complex and may include different actions like licking, nibbling or scratching (see also S1 Table), the resulting behaviour class probably includes a broad range of characteristic summary statistics and may thus be more easily confused with other behaviour classes.

Lastly, we cannot consider natural behaviours that usually are not or very rarely observed in zoo-kept animals such as mating or territorial behaviour. These behaviours (as well as other, unknown behaviours) might very well have occurred in the wild where they could not have been classified because the algorithms were not trained on them. As possible for the mixed bursts, these untrained behaviours could be detected through the probability threshold and classified as “other”.

Another cause of the poor performance of the SVM and RF in predicting wild fox behaviour could be the small sample size of the data set of the captive foxes. With more observational data of the captive foxes, we might have been able to train a more robust model. An ideal model would thus be trained on more data. However, many studies in the field of wildlife ecology and conservation research face the problem of small sample sizes. Species may either be hard to observe or assess (e.g. due to remote or impassable habitats or an elusive nature of the species) or simply by cause of being too rarely distributed and/or barely zoo-kept. By introducing the moving window approach to wildlife behaviour recognition, we may actually have found a promising possibility to deal with these challenging conditions.

### The artificial neural network (ANN)

The ANN showed comparable results to SVM and RF in model validation and consequently to the literature as cited above. However, when applying it to the same wild individuals the SVM and RF failed on, it classified a very different set of behaviours (Table 3). If we had only tested the two established approaches, we would have concluded that the transfer of a behaviour classification model trained on captive foxes to wild individuals is not possible. In contrast, the ANN with a moving window shows promising results that hopefully prompt further investigation into its potential use for wildlife ecology. The approach brings two additional advantages, besides the increased sample size: First, the likely better handling of recordings with mixed behaviours: By reducing the number of measurements per burst, the proportion of a potential second behaviour in the same burst is reduced. In some cases, this reduction may be enough to calculate similar summary statistics to a burst with only one of the behaviours. Second, the introduction of an ensemble learning effect: In case of a specific behaviour being performed in an unusual way or a burst containing more than one behaviour, it will be harder to infer the correct behaviour. As the moving window creates 32 subsamples of the original burst, using a majority vote for the resulting 32 assigned behaviours can reduce the uncertainty of the assignments. This framework could be a useful tool for future studies in wildlife research, especially for the study of species that are rarely kept in zoos or the study of behaviours that are hard to observe even in captivity.

The best size for the moving window was determined based on the maximal performance and slope of the GAM fit in the simulation plot (Fig 3). Our aim was to find a window size with a high mean performance that is small enough for generating sufficient data. Unfortunately, reducing the window size was found to negatively impact model performance [15]. Performance seemed to be at its maximum at window size 79. Larger windows would result in fewer subsets at worse performance, while performance also decreased for window sizes smaller than 79. Considering the slope of the GAM fit, the performance changed only marginally at window sizes 78 and 80 compared to 79. Therefore, we expect the model to perform similarly well at these window sizes. We suggest considering the slope because this approach may not always show a clear maximum like in our case. In cases of multiple maxima or plateau formations, the slope will help to inform on the smallest window size with the best performance.

A problem that may occur with the moving window approach is incorrect classification through overfitting [41]. By creating several similar subsets of the same burst, the ANN could build a model that fits the training data too well, i.e. even slight differences between training data and new data of the same behaviour class would result in the classification of different behaviours, with an overfitted model. Variation within the behaviour classes could also cause incorrect classifications if a single behaviour is realised outside the normal variation. There is no clear method to distinguish between these two causes of incorrect classifications. However, in the following we discuss the credibility of the assigned wild fox behaviour and argue that the moving window approach does not introduce overfitting.

### Output credibility

The classification results of the classic methods appear to be obviously incorrect. At first glance, the output of the ANN appears more plausible than the output of RF and SVM, on account of all six behaviours getting classified. Still, the actual accuracy cannot be determined as wild individuals could not be observed. Since this may be true for most tagged wildlife, we provide four strategies to indirectly assess the credibility of the ANN output.

When looking at the time-dependent composition of behaviours, they appear quite consistent over individuals and time. Generally, some variation between individuals is apparent and some behaviour events seem to be misclassified. Individual differences in moving behaviour, for example, may result in the assignment of either walking or trotting when the algorithm is not accurate enough. However, an overall pattern is evident for most of the foxes and the temporal distribution appears plausible: The ANN output suggests that the foxes predominantly rest during the day and are active at night as well as during twilight (Fig 4, S1–S8 Figs) which corresponds well with described nocturnal-crepuscular activity patterns of red foxes (e.g. [42, 43]).

There also seem to be seasonal changes in these patterns, with fewer resting events during dark hours in the summer months. Although there are only two complete year-round datasets available, this pattern appears reasonable as the nights in summertime at this longitude are much shorter than during winter months (in Berlin, the daily dark period ranges from 7 to 17 hours during the course of a year). Thus, foxes should use the full night-time spectrum in summer for their activities, while during winter the higher availability of potential activity time allows nocturnal resting events. This behavioural plasticity in activity has also been shown by Ricci et al. [44] who found that foxes are active in different zones and hours of the day, according to the season.

Like trotting and walking, feeding is mostly classified at night-time. Feeding events occur in no clustered manner. The mixture of movement and feeding events reflects the feeding ecology of foxes which do not feed on large prey. Foxes mostly hunt for small prey like mice and voles and often rather scavenge than hunt, especially in urbanized areas [45, 46].

While the behaviour composition is very similar across all individuals we cannot rule out the possibility that the wild foxes performed a behaviour that is very similar to another behaviour class that we did not observe in the captive foxes. Consequently this behaviour class would have been misclassified as one of the behaviour classes that we did observe in the captive foxes. Due to the nature of our study we do not have the possibility to test whether that was the case or not because we do not have access to the actual behaviour of the wild foxes. In any case, this behaviour class or classes would have to be behaviour that are universal in wild foxes but are not or very rarely performed by captive foxes. Obtaining observation and acceleration data of such behaviour for the training of any machine learning algorithm would be quite challenging. Generally, similar results do necessarily indicate correct predictions, but dissimilarities between individuals could hint to poor model performance. Anyhow, the credibility of the output has to be addressed by using different approaches. Therefore we also used GPS data to relate the occurrence of GPS-clusters as well as GPS-based speed values to the assigned behaviour classes.

In particular, we focused on resting and movement behaviour (trotting and walking), with an obvious connection to be expected. Resting as a stationary behaviour should get classified predominantly at locations where GPS points are clustered (Fig 5). We found that 38% to 74% of resting bursts were located within such a cluster. The remaining bursts may reflect cases when the individual had just temporarily stopped moving. Standing still or sitting briefly during an active phase would also be classified as resting but may not be associated with a GPS-cluster.

Trotting as a locomotive behaviour was expected to show low association with GPS-clusters and was only classified at a cluster for 2% to 9% of all bursts (Fig 5, S8–S14 Figs). As foxes move away from or to a resting site, it is reasonable for some trotting to be classified within GPS clusters. The analysis did not target feeding, caching or grooming, as those behaviours can be performed in a clustered or non-clustered manner. The analysis of behaviour assignments in relation to speed shows a reverse picture: Behaviours that show a weak association with GPS clusters show a higher speed and vice versa (S8–S14 Figs). However, we could use only 5% to 15% of all data for the speed analysis (S6 Table). The method described here is hence more applicable when the recording of location and acceleration data is better synchronised.

Finally, we analysed the ODBA, an indicator of body movement [18] that was shown to correspond well with the activity level of specific behaviours [15]. When we compared the temporal distribution of ODBA values to that of the classified trotting and resting events, we saw an association of high ODBA values and trotting behaviour and low ODBA values and resting behaviour, respectively. Again, the nocturnal-crepuscular activity pattern was visible (Fig 6, S15–S21 Figs).

While the above mentioned examples appear conceivable, the interpretation of some behaviours may be puzzling, and their biological credibility is difficult to gauge. For instance, we could not identify any pattern for caching behaviour, and grooming seems to be generally over-classified. Its complexity and the resulting variability in the training data set may increase misclassification of unknown behaviours, especially when considering that six behaviours do not represent the full variety of behaviour that this mobile carnivore displays in the wild. The latter is clearly more significant for fast-moving animals like the highly agile red fox that displays a variety of complex movement patterns. Generating as precise outputs as they, for example, have been shown for grazing animals with their limited body flexibility and behavioural repertoire (see [28, 47]), therefore remains a challenge. In another study on captive red foxes Painter et al. [48] could classify three exhibited behaviours with an accuracy of 95.7% when training on one individual, and predicted the behaviour of a second individual at an accuracy of 66.7%, suggesting that the classifier can extract behaviours across multiple foxes.

In the present study, a broader training dataset of more captive individuals could possibly improve the output of the ANN for the wild foxes and permit more precise recognition of specific behaviours. However, our results suggest that the behaviour inferred by the ANN corresponds well with the actual behaviour of the logged foxes. Despite some unsolved issues, the ANN thus seems to be a promising approach to infer wildlife behaviour, even in cases where methods suggested by existing literature fail.

## Conclusion

We here compare the relative predictive power of different machine learning approaches in inferring wildlife behaviour and we could show that good results for the validation of the models will not necessarily lead to good results when these models are applied in the field. We provide a framework to use acceleration data and an Artificial Neural Network to infer the behaviour of wild foxes, using a training data set obtained from captive individuals. We also present four strategies to address the plausibility of such behaviour classification output when no direct validation is possible. Although not all validation strategies may be applicable for every species, this framework should not be restricted to the studied species. The successful application of the ANN for behavioural classification on field data offers exciting potential to study the behaviour of animals in the wild without direct observation.

## Supporting information

S1 Fig Time-dependent composition of behaviours of fox “Gisel”. Stacked bars represent the proportion of each behaviour at a given time of day, in each month. The data shown here span from September 2016 until March 2017. Plots for September and March are only based on 2 and 7 days, respectively, and cannot be interpreted. Because of a logger failure the February plot is only based on 12 days and should also not be considered. Gisel shows more walking and feeding than most other individuals.
(TIF)

S2 Fig Time-dependent composition of behaviours of fox “Hazel”. Stacked bars represent the proportion of each behaviour at a given time of day, in each month. The data shown here span from October 2016 until February 2017. Hazel shows more feeding than most other individuals.
(TIF)

S3 Fig Time-dependent composition of behaviours of fox “Ida”. Stacked bars represent the proportion of each behaviour at a given time of day, in each month. The data shown here span from November 2016 until April 2017. Ida shows almost no walking.
(TIF)

S4 Fig Time-dependent composition of behaviours of fox “Jack”. Stacked bars represent the proportion of each behaviour at a given time of day, in each month. The data shown here span from January 2017 until December 2017. The plot for January is only based on 7 days and should not be interpreted. Jack shows much more grooming than all other individuals, as well as much less resting.
(TIF)

S5 Fig Time-dependent composition of behaviours of fox “Kyna”. Stacked bars represent the proportion of each behaviour at a given time of day, in each month. The data shown here span from February 2017 until November 2017. The logger failed between the 15th of June and the 26 of July. The June plot looks jagged because of the resulting lack of data. The July plot cannot be interpreted because it only relies on 6 days. Generally, less trotting is predicted for Kyna compared to the other foxes.
(TIF)

S6 Fig Time-dependent composition of behaviours of fox “Nikita”. Stacked bars represent the proportion of each behaviour at a given time of day, in each month. The data shown here span from September 2017 until January 2018. The September and January plots cannot be interpreted because both are based on 5 days only. For this fox much more walking than trotting is predicted. In addition, much more feeding is predicted than for most other individuals.
(TIF)

S7 Fig Time-dependent composition of behaviours of fox “Porthos”. Stacked bars represent the proportion of each behaviour at a given time of day, in each month. The data shown here span from October 2017 until June 2018. The logger failed for 15 days in March and for 8 days in April. The March plot therefore shows only half as much data as the other plots and looks more jagged. The October plot is only based on 6 days and can thus not be interpreted properly.
(TIF)

S8 Fig Behaviour assignments of “Gisel” in relation to GPS clusters (A) and speed (B). The numbers in the brackets indicate the sample size of each behaviour class. (A) Resting shows the highest association with GPS clusters (57%) and trotting the lowest (2%). (B) Behaviour prediction in relation to speed. Resting appears at significantly lower speeds than trotting (Wilcoxon rank sum test, W = 727995, p < 0.001).
(TIF)

S9 Fig Behaviour assignments of “Hazel” in relation to GPS clusters (A) and speed (B). The numbers in the brackets indicate the sample size of each behaviour class. (A) Resting shows the highest association with GPS clusters (38%) and trotting the lowest (9%). Resting is predicted much less in clusters than in most other study foxes. (B) Behaviour prediction in relation to speed. Resting appears at significantly lower speeds than trotting (Wilcoxon rank sum test, W = 489223, p < 0.001).
(TIF)

S10 Fig Behaviour assignments of “Ida” in relation to GPS clusters (A) and speed (B). The numbers in the brackets indicate the sample size of each behaviour class. (A) Resting shows the highest association with GPS clusters (46%) and trotting the lowest (9%). Resting is predicted much less in clusters than in most other study foxes. (B) Behaviour prediction in relation to speed. Resting appears at significantly lower speeds than trotting (Wilcoxon rank sum test, W = 803283, p < 0.001).
(TIF)

S11 Fig Behaviour assignments of “Jack” in relation to GPS clusters (A) and speed (B). The numbers in the brackets indicate the sample size of each behaviour class. (A) Resting shows the highest association with GPS clusters (74%) and trotting the lowest (6%). Resting is predicted much less in clusters than in most other study foxes. (B) Behaviour prediction in relation to speed. Resting appears at significantly lower speeds than trotting (Wilcoxon rank sum test, W = 295149, p < 0.001).
(TIF)

S12 Fig Behaviour assignments of “Kyna” in relation to GPS clusters (A) and speed (B). The numbers in the brackets indicate the sample size of each behaviour class. (A) Resting shows the highest association with GPS clusters (57%) and trotting the lowest (3%). (B) Behaviour prediction in relation to speed. Resting appears at significantly lower speeds than trotting (Wilcoxon rank sum test, W = 428274, p < 0.001).
(TIF)

S13 Fig Behaviour assignments of “Nikita” in relation to GPS clusters (A) and speed (B). The numbers in the brackets indicate the sample size of each behaviour class. (A) Resting shows the highest association with GPS clusters (69%) and trotting the lowest (8%). (B) Behaviour prediction in relation to speed. Resting appears at significantly lower speeds than trotting (Wilcoxon rank sum test, W = 327626, p < 0.001).
(TIF)

S14 Fig Behaviour assignments of “Porthos” in relation to GPS clusters (A) and speed (B). The numbers in the brackets indicate the sample size of each behaviour class. (A) Resting shows the highest association with GPS clusters (68%) and trotting the lowest (8%). (B) Behaviour prediction in relation to speed. Resting appears at significantly lower speeds than trotting (Wilcoxon rank sum test, W = 4454986, p < 0.001).
(TIF)

S15 Fig Temporal distribution of trotting (A), (B) resting and (C) ODBA values of “Gisel”. The red lines indicate the sunset and sunrise. (A) Black spaces indicate times at which trotting behaviour was predicted whereas white spaces indicate assignments of all other behaviours. Trotting is predominantly predicted during the night. (B) Black spaces indicate times at which resting behaviour was predicted whereas white spaces indicate assignments of all other behaviours. Resting is predominantly predicted during the day. The switch between trotting, resting and other behaviours is mostly oriented at the sunset and sunrise. (C) Higher ODBA values are indicated by darker spaces. ODBA values are higher at night than during daytime. Discontinuities in the red line are caused by missing data due to the logger not recording data at the time.
(TIF)

S16 Fig Temporal distribution of trotting (A), (B) resting and (C) ODBA values of “Hazel”. The red lines indicate the sunset and sunrise. (A) Black spaces indicate times at which trotting behaviour was predicted whereas white spaces indicate assignments of all other behaviours. Trotting is predominantly predicted during the night. (B) Black spaces indicate times at which resting behaviour was predicted whereas white spaces indicate assignments of all other behaviours. Resting is predominantly predicted during the day. The switch between trotting, resting and other behaviours is mostly oriented at the sunset and sunrise. (C) Higher ODBA values are indicated by darker spaces. ODBA values are higher at night than during daytime.
(TIF)

S17 Fig Temporal distribution of trotting (A), (B) resting and (C) ODBA values of “Ida”. The red lines indicate the sunset and sunrise. (A) Black spaces indicate times at which trotting behaviour was predicted whereas white spaces indicate assignments of all other behaviours. Trotting is predominantly predicted during the night. (B) Black spaces indicate times at which resting behaviour was predicted whereas white spaces indicate assignments of all other behaviours. Resting is predominantly predicted during the day. The switch between trotting, resting and other behaviours is mostly oriented at the sunset and sunrise. (C) Higher ODBA values are indicated by darker spaces. ODBA values are higher at night than during daytime.
(TIF)

S18 Fig Temporal distribution of trotting (A), (B) resting and (C) ODBA values of “Jack”. The red lines indicate the sunset and sunrise. (A) Black spaces indicate times at which trotting behaviour was predicted whereas white spaces indicate assignments of all other behaviours. Trotting is predominantly predicted during the night. (B) Black spaces indicate times at which resting behaviour was predicted whereas white spaces indicate assignments of all other behaviours. Resting is predominantly predicted during the day. The switch between trotting, resting and other behaviours is mostly oriented at the sunset and sunrise. (C) Higher ODBA values are indicated by darker spaces. ODBA values are higher at night than during daytime.
(TIF)

S19 Fig Temporal distribution of trotting (A), (B) resting and (C) ODBA values of “Kyna”. The red lines indicate the sunset and sunrise. (A) Black spaces indicate times at which trotting behaviour was predicted whereas white spaces indicate assignments of all other behaviours. Trotting is predominantly predicted during the night. (B) Black spaces indicate times at which resting behaviour was predicted whereas white spaces indicate assignments of all other behaviours. Resting is predominantly predicted during the day. The switch between trotting, resting and other behaviours is mostly oriented at the sunset and sunrise. (C) Higher ODBA values are indicated by darker spaces. ODBA values are higher at night than during daytime. Discontinuities in the red line are caused by missing data due to the logger not recording data at the time.
(TIF)

S20 Fig Temporal distribution of trotting (A), (B) resting and (C) ODBA values of “Nikita”. The red lines indicate the sunset and sunrise. (A) Black spaces indicate times at which trotting behaviour was predicted whereas white spaces indicate assignments of all other behaviours. Trotting is predominantly predicted during the night. (B) Black spaces indicate times at which resting behaviour was predicted whereas white spaces indicate assignments of all other behaviours. Resting is predominantly predicted during the day. The switch between trotting, resting and other behaviours is mostly oriented at the sunset and sunrise. (C) Higher ODBA values are indicated by darker spaces. ODBA values are higher at night than during daytime.
(TIF)

S21 Fig Temporal distribution of trotting (A), (B) resting and (C) ODBA values of “Porthos”. The red lines indicate the sunset and sunrise. (A) Black spaces indicate times at which trotting behaviour was predicted whereas white spaces indicate assignments of all other behaviours. Trotting is predominantly predicted during the night. (B) Black spaces indicate times at which resting behaviour was predicted whereas white spaces indicate assignments of all other behaviours. Resting is predominantly predicted during the day. The switch between trotting, resting and other behaviours is mostly oriented at the sunset and sunrise. (C) Higher ODBA values are indicated by darker spaces. ODBA values are higher at night than during daytime. Discontinuities in the red line are caused by missing data due to the logger not recording data at that time.
(TIF)

S1 Table Ethogram for the captive foxes—Modified from [16]. Behavioural observations were restricted to the listed categories. Each burst could only have one behaviour assigned. Observations not matching any description as well as observations of more than one behaviour per burst were excluded from the analysis.
(DOCX)

S2 Table List of all predictors and how they were calculated. References refer to when the respective predictor was first introduced in the context of behaviour prediction. All predictors except the Fast Fourier Transformation can be considered summary statistics because they result in a single number. We add the complete Fast Fourier Spectrum as predictors. The total amount of predictors from the Fast Fourier Spectrum is therefore dependant on burst length.
(DOCX)

S3 Table Confusion matrix for the support vector machine (SVM) validation. Columns show expected behaviours known from observation, rows show behaviours assigned by the SVM. Values on the diagonal (bold) represent behaviours assigned correctly. All values off the diagonal are incorrect assignments that show which behaviours were confused with each other (for example 16 events of feeding were incorrectly classified as grooming).
(DOCX)

S4 Table Confusion matrix for the random forest (RF) validation. Columns show expected behaviours known from observation, rows show behaviours assigned by the RF. Values on the diagonal (bold) represent behaviours assigned correctly. All values off the diagonal are incorrect assignments that show which behaviours were confused with each other (e.g. 17 events of feeding were incorrectly classified as grooming).
(DOCX)

S5 Table Confusion matrix for the artificial neural network (ANN) validation. Columns show expected behaviours known from observation, rows show behaviours assigned by the ANN. Values on the diagonal (bold) represent behaviours assigned correctly. All values off the diagonal are incorrect assignments that show which behaviours were confused with each other (for example 15 events of resting were incorrectly predicted as feeding).
(DOCX)

S6 Table Proportion of data used for the speed analysis. Most of the times GPS and acceleration data were not recorded simultaneously. For the speed analysis we considered only acceleration data that was recorded within 10 seconds of a GPS recording.
(DOCX)
