# Supplementary figures and images for: Machine learning goes wild: Using data from captive individuals to infer wildlife behaviours
Source: PLoS One. 2020 May 5;15(5):e0227317. doi: 10.1371/journal.pone.0227317 (PMC7200095; doi:10.1371/journal.pone.0227317)

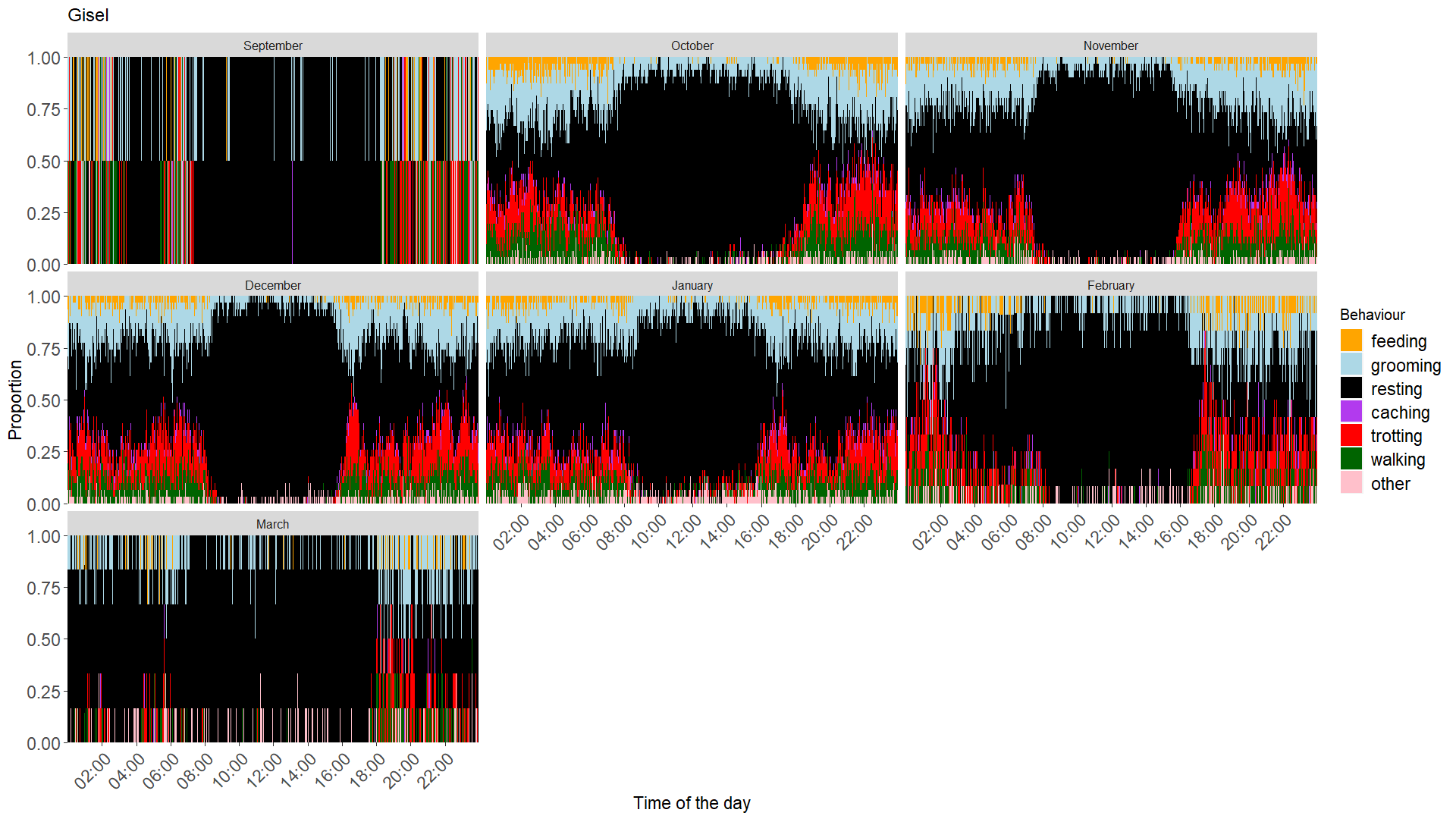

Supplement: S1 Fig — Stacked bars represent the proportion of each behaviour at a given time of day, in each month. The data shown here span from September 2016 until March 2017. Plots for September and March are only based on 2 and 7 days, respectively, and cannot be interpreted. Because of a logger failure the February plot is only based on 12 days and should also not be considered. Gisel shows more walking and feeding than most other individuals. (TIF) [file pone.0227317.s001.tif]

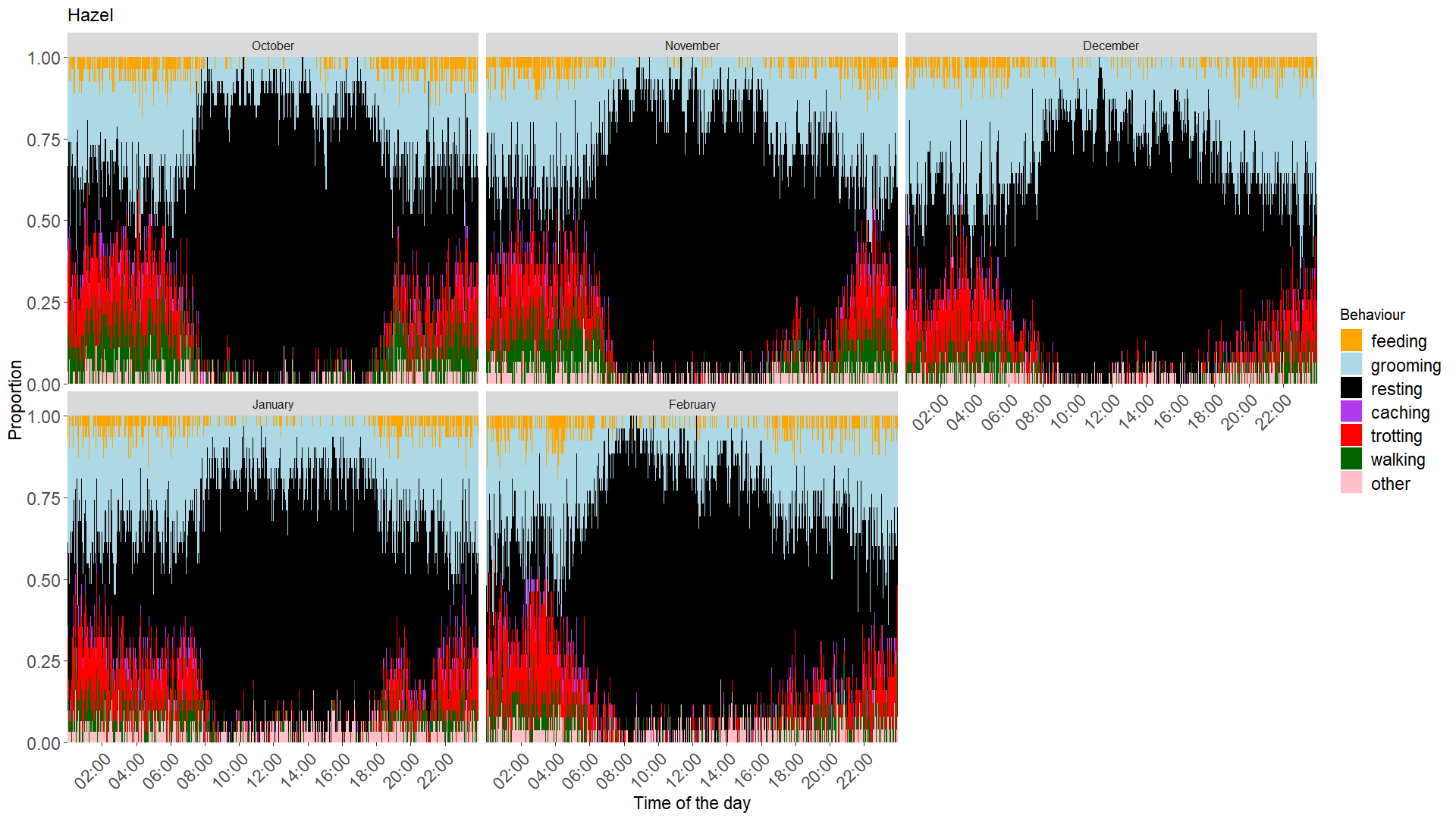

Supplement: S2 Fig — Stacked bars represent the proportion of each behaviour at a given time of day, in each month. The data shown here span from October 2016 until February 2017. Hazel shows more feeding than most other individuals. (TIF) [file pone.0227317.s002.tif]

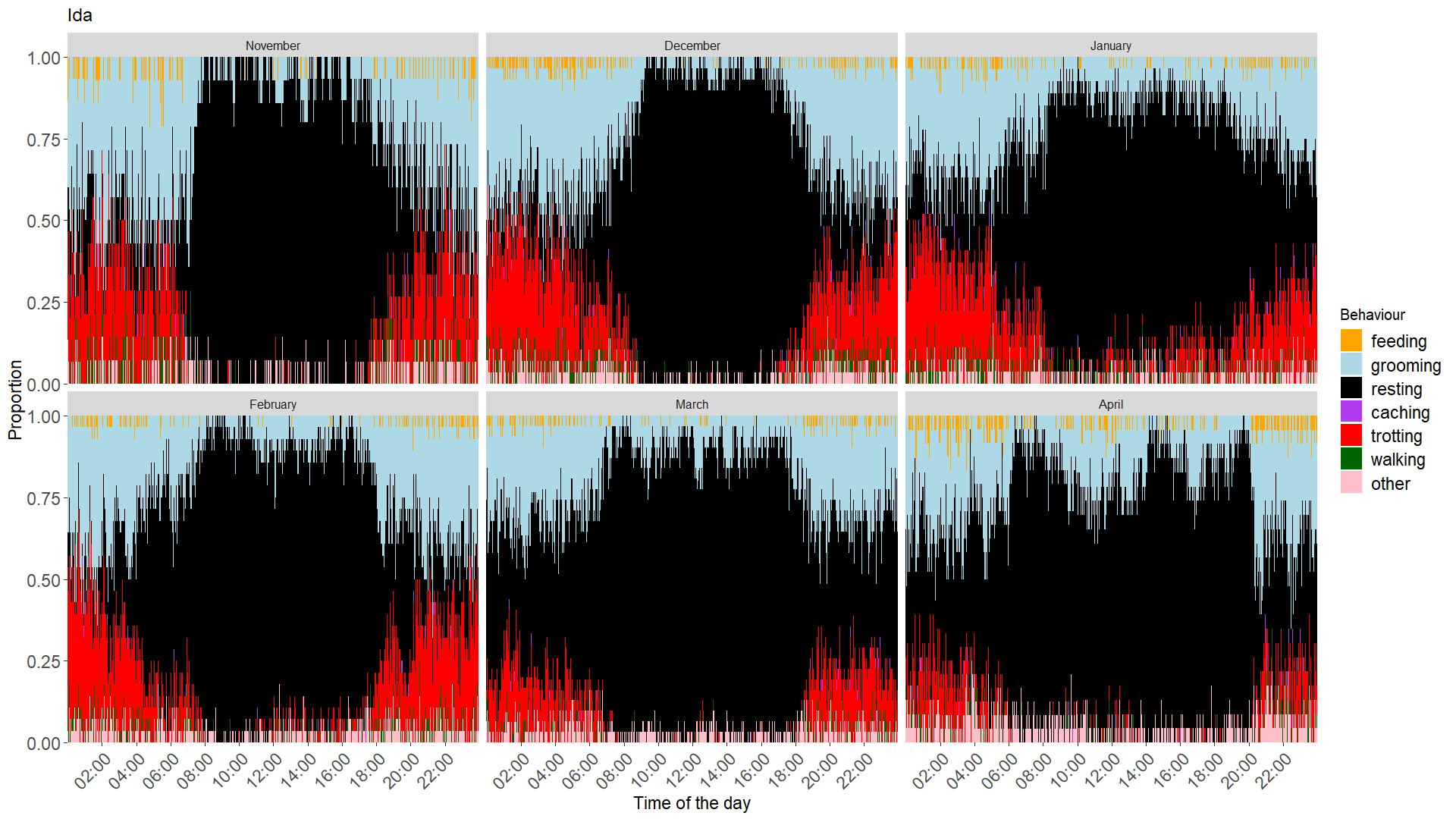

Supplement: S3 Fig — Stacked bars represent the proportion of each behaviour at a given time of day, in each month. The data shown here span from November 2016 until April 2017. Ida shows almost no walking. (TIF) [file pone.0227317.s003.tif]

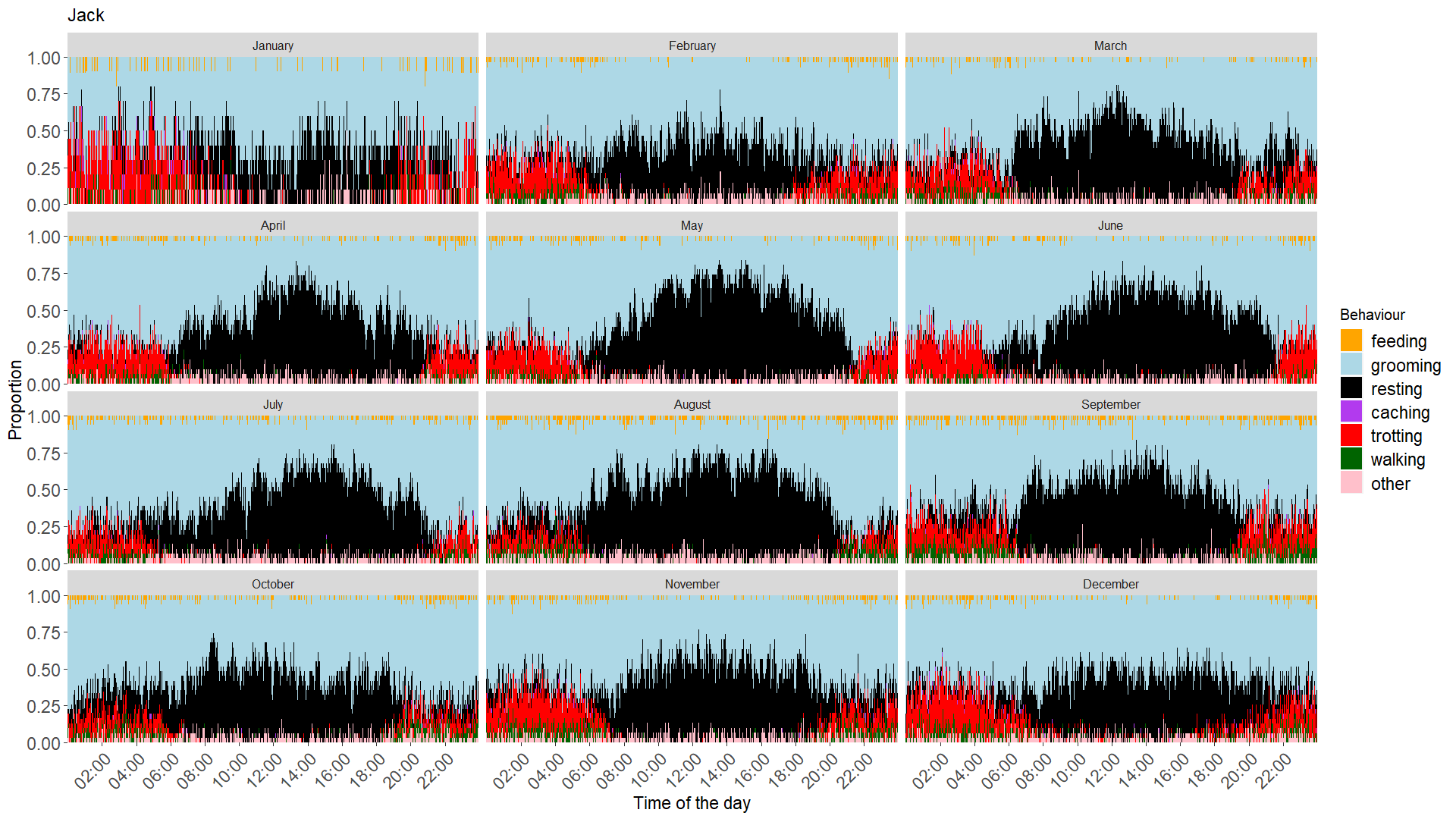

Supplement: S4 Fig — Stacked bars represent the proportion of each behaviour at a given time of day, in each month. The data shown here span from January 2017 until December 2017. The plot for January is only based on 7 days and should not be interpreted. Jack shows much more grooming than all other individuals, as well as much less resting. (TIF) [file pone.0227317.s004.tif]

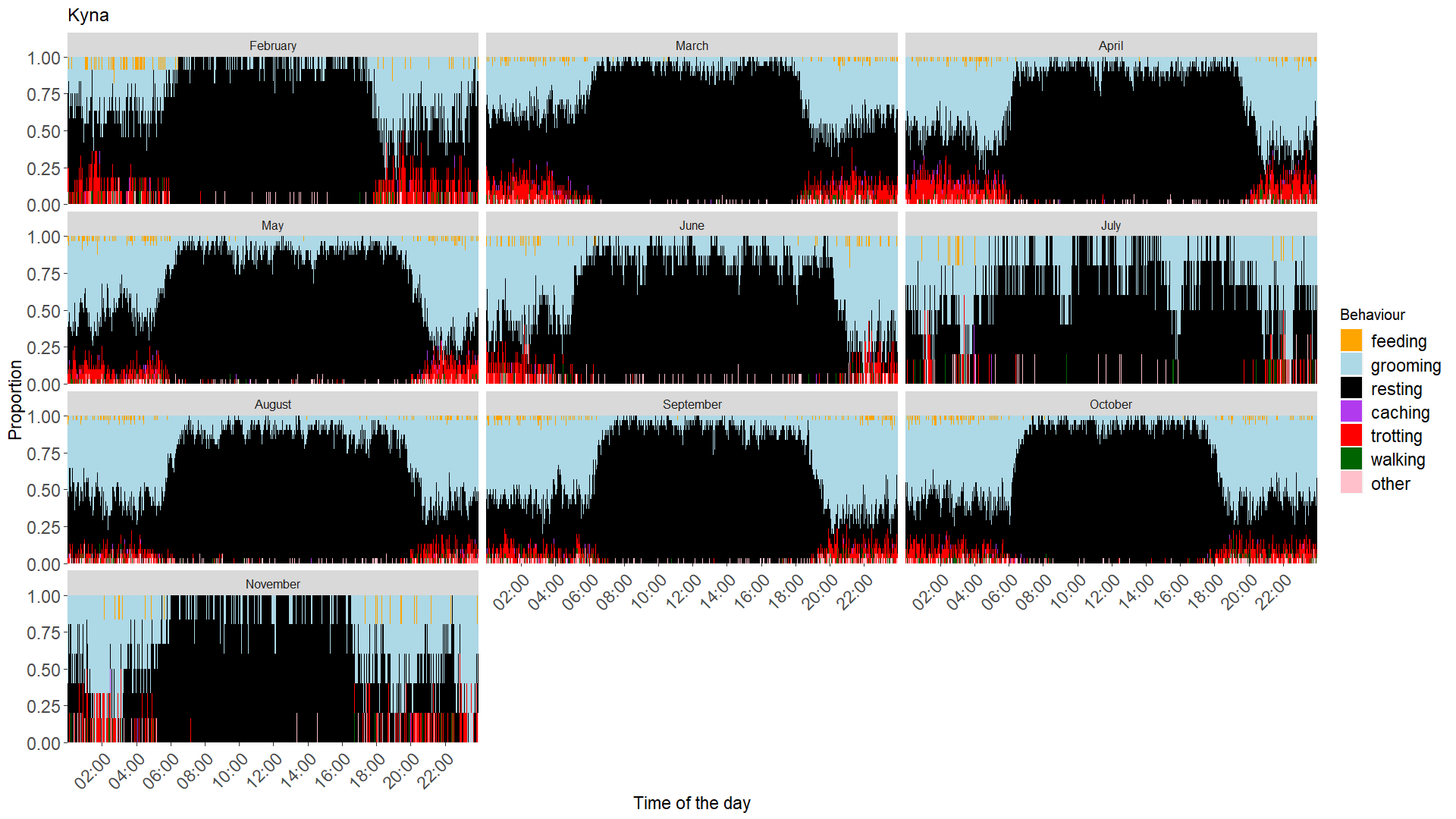

Supplement: S5 Fig — Stacked bars represent the proportion of each behaviour at a given time of day, in each month. The data shown here span from February 2017 until November 2017. The logger failed between the 15th of June and the 26 of July. The June plot looks jagged because of the resulting lack of data. The July plot cannot be interpreted because it only relies on 6 days. Generally, less trotting is predicted for Kyna compared to the other foxes. (TIF) [file pone.0227317.s005.tif]

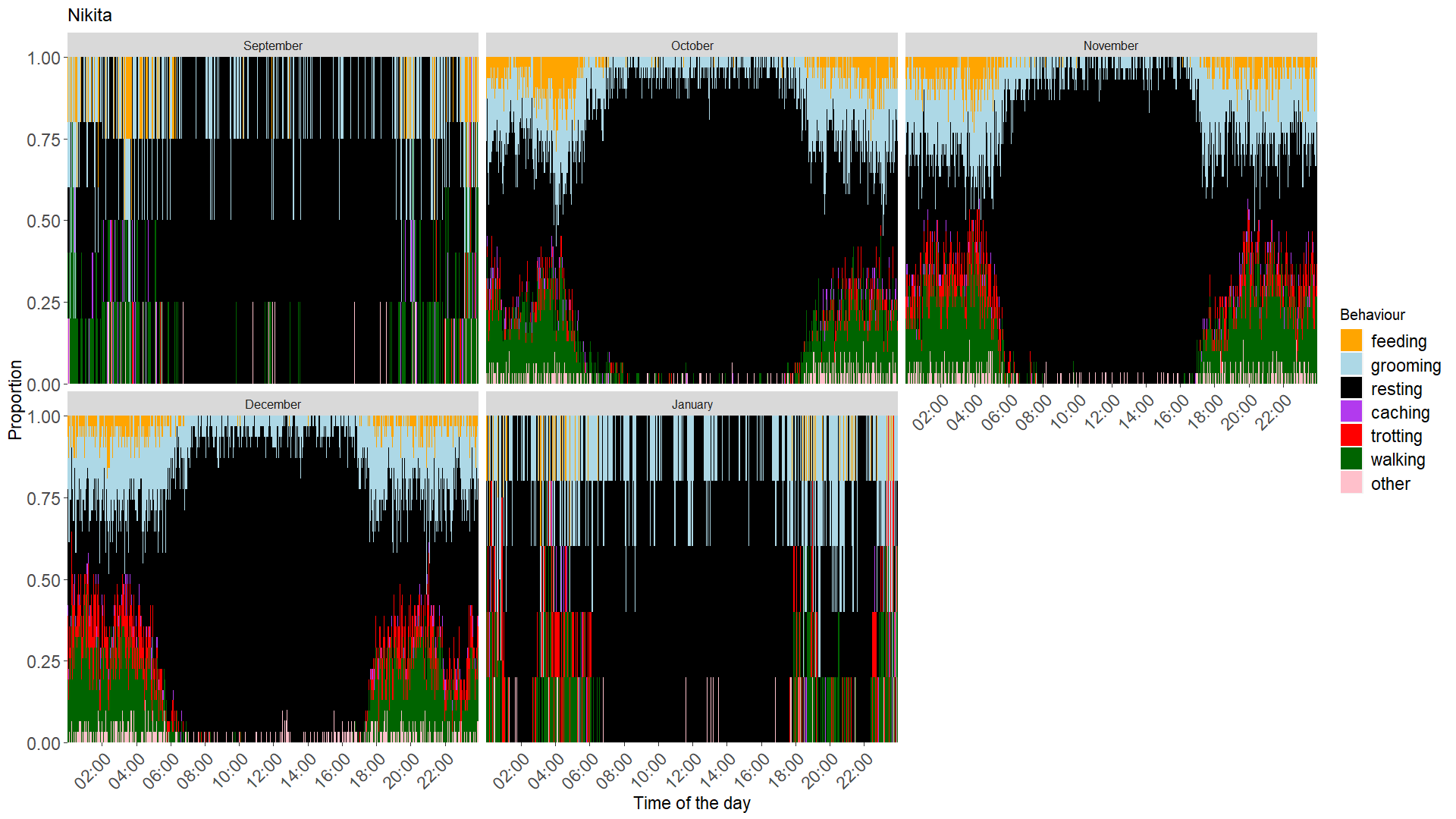

Supplement: S6 Fig — Stacked bars represent the proportion of each behaviour at a given time of day, in each month. The data shown here span from September 2017 until January 2018. The September and January plots cannot be interpreted because both are based on 5 days only. For this fox much more walking than trotting is predicted. In addition, much more feeding is predicted than for most other individuals. (TIF) [file pone.0227317.s006.tif]

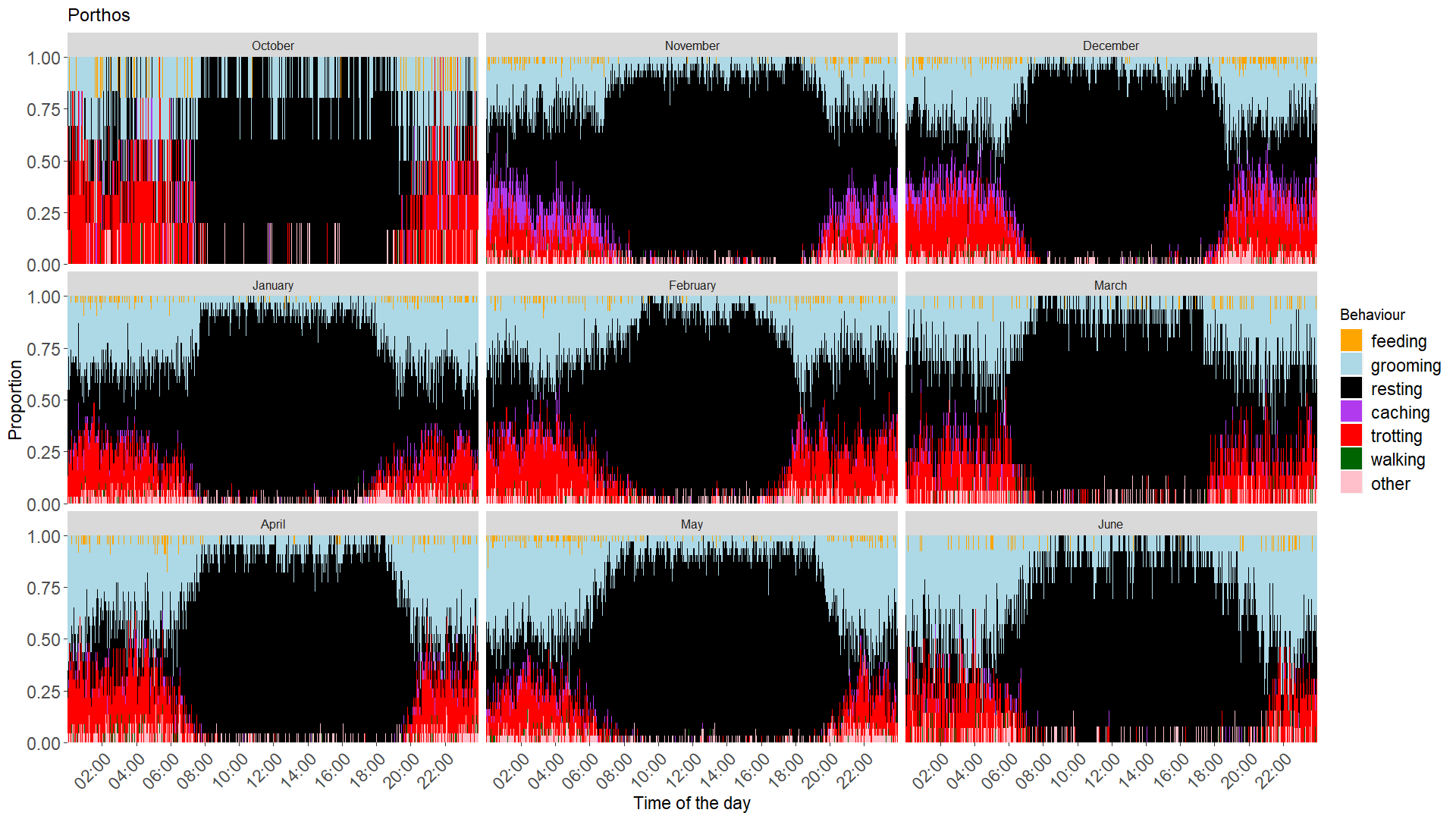

Supplement: S7 Fig — Stacked bars represent the proportion of each behaviour at a given time of day, in each month. The data shown here span from October 2017 until June 2018. The logger failed for 15 days in March and for 8 days in April. The March plot therefore shows only half as much data as the other plots and looks more jagged. The October plot is only based on 6 days and can thus not be interpreted properly. (TIF) [file pone.0227317.s007.tif]

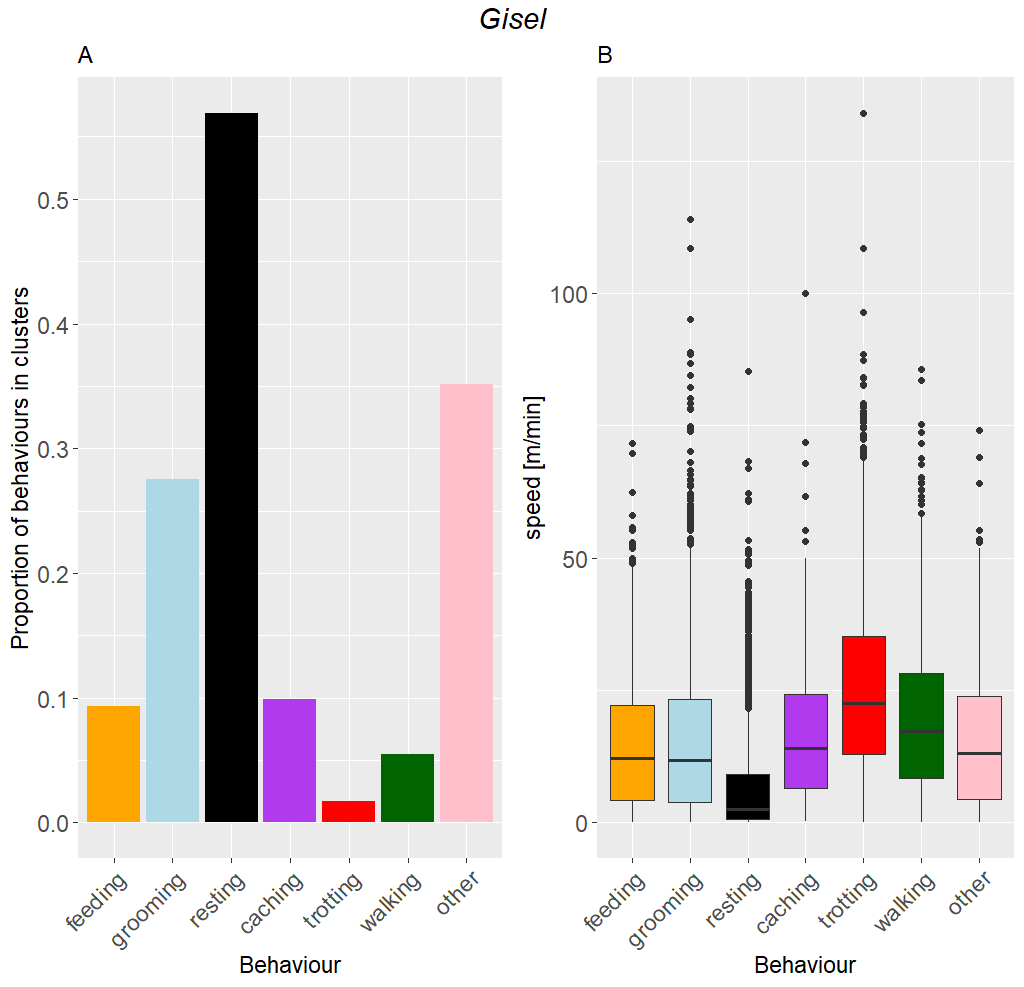

Supplement: S8 Fig — The numbers in the brackets indicate the sample size of each behaviour class. (A) Resting shows the highest association with GPS clusters (57%) and trotting the lowest (2%). (B) Behaviour prediction in relation to speed. Resting appears at significantly lower speeds than trotting (Wilcoxon rank sum test, W = 727995, p < 0.001). (TIF) [file pone.0227317.s008.tif]

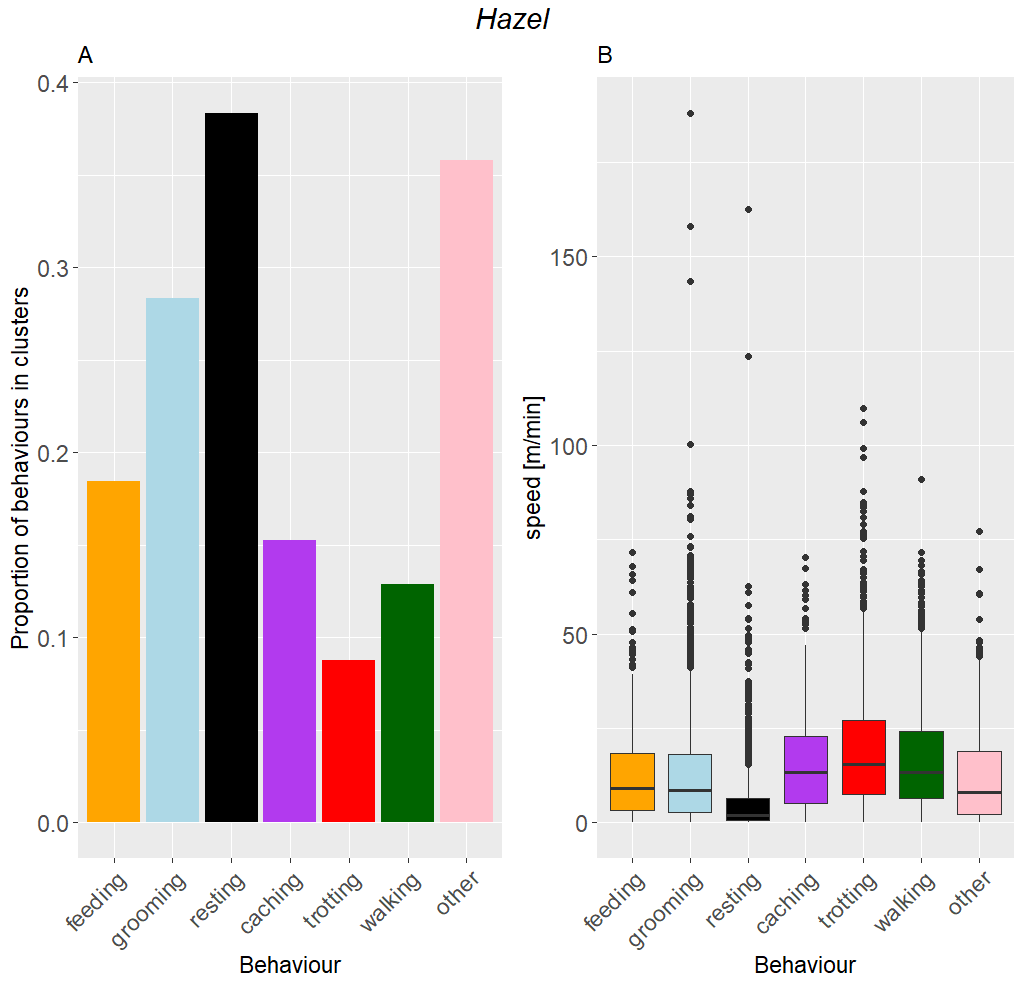

Supplement: S9 Fig — The numbers in the brackets indicate the sample size of each behaviour class. (A) Resting shows the highest association with GPS clusters (38%) and trotting the lowest (9%). Resting is predicted much less in clusters than in most other study foxes. (B) Behaviour prediction in relation to speed. Resting appears at significantly lower speeds than trotting (Wilcoxon rank sum test, W = 489223, p < 0.001). (TIF) [file pone.0227317.s009.tif]

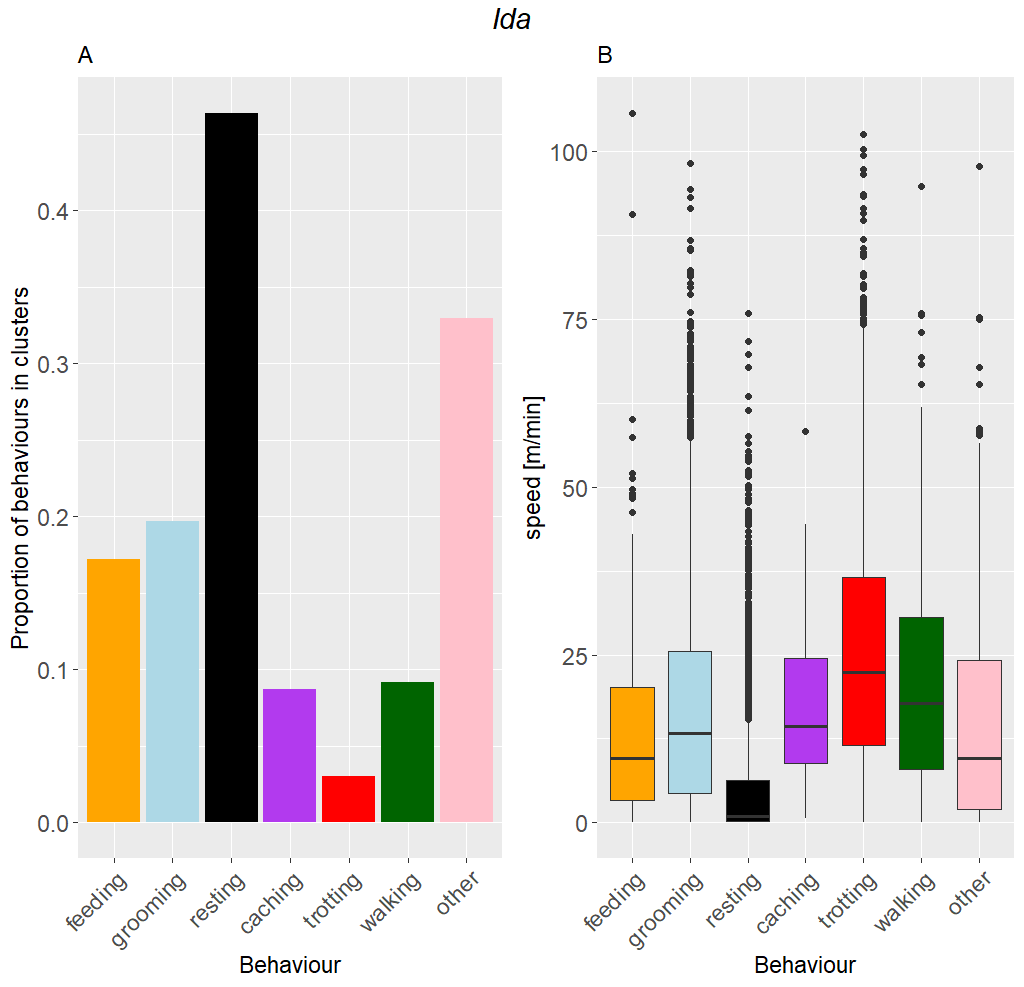

Supplement: S10 Fig — The numbers in the brackets indicate the sample size of each behaviour class. (A) Resting shows the highest association with GPS clusters (46%) and trotting the lowest (9%). Resting is predicted much less in clusters than in most other study foxes. (B) Behaviour prediction in relation to speed. Resting appears at significantly lower speeds than trotting (Wilcoxon rank sum test, W = 803283, p < 0.001). (TIF) [file pone.0227317.s010.tif]

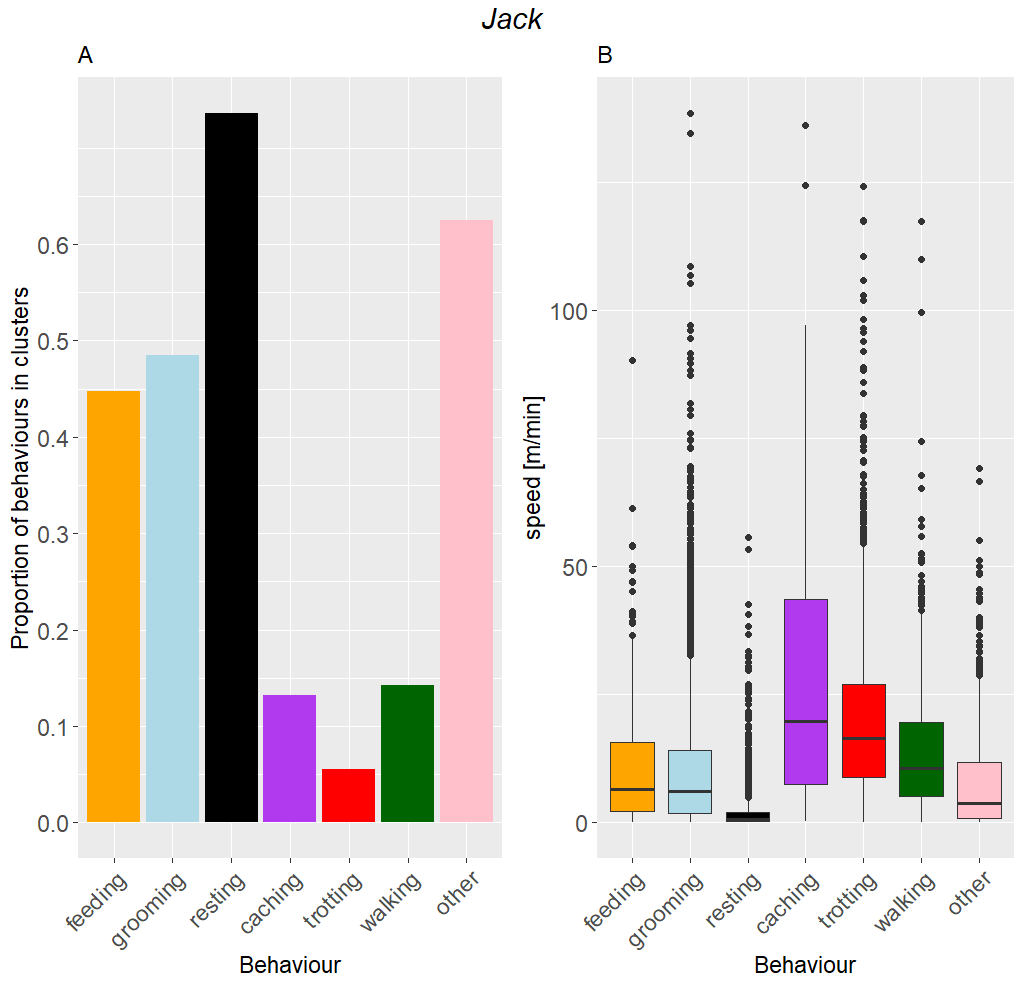

Supplement: S11 Fig — The numbers in the brackets indicate the sample size of each behaviour class. (A) Resting shows the highest association with GPS clusters (74%) and trotting the lowest (6%). Resting is predicted much less in clusters than in most other study foxes. (B) Behaviour prediction in relation to speed. Resting appears at significantly lower speeds than trotting (Wilcoxon rank sum test, W = 295149, p < 0.001). (TIF) [file pone.0227317.s011.tif]

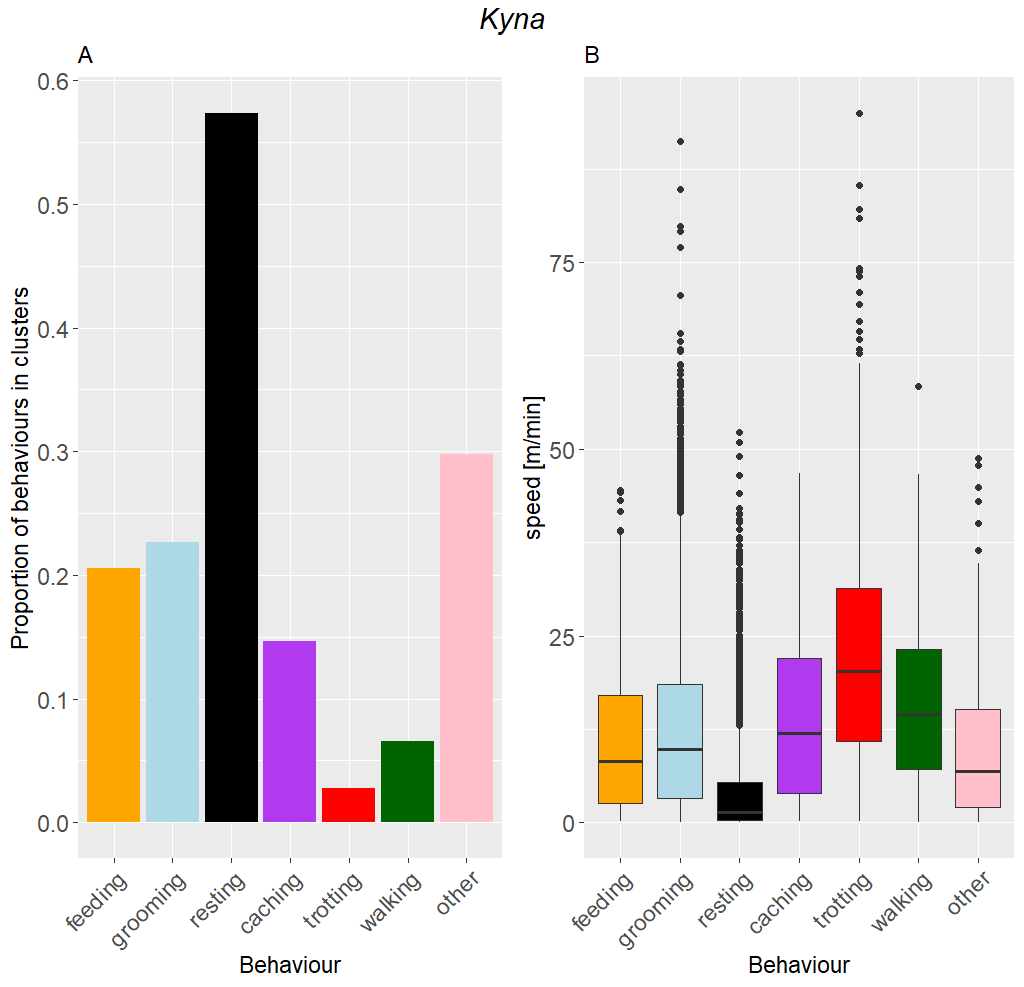

Supplement: S12 Fig — The numbers in the brackets indicate the sample size of each behaviour class. (A) Resting shows the highest association with GPS clusters (57%) and trotting the lowest (3%). (B) Behaviour prediction in relation to speed. Resting appears at significantly lower speeds than trotting (Wilcoxon rank sum test, W = 428274, p < 0.001). (TIF) [file pone.0227317.s012.tif]

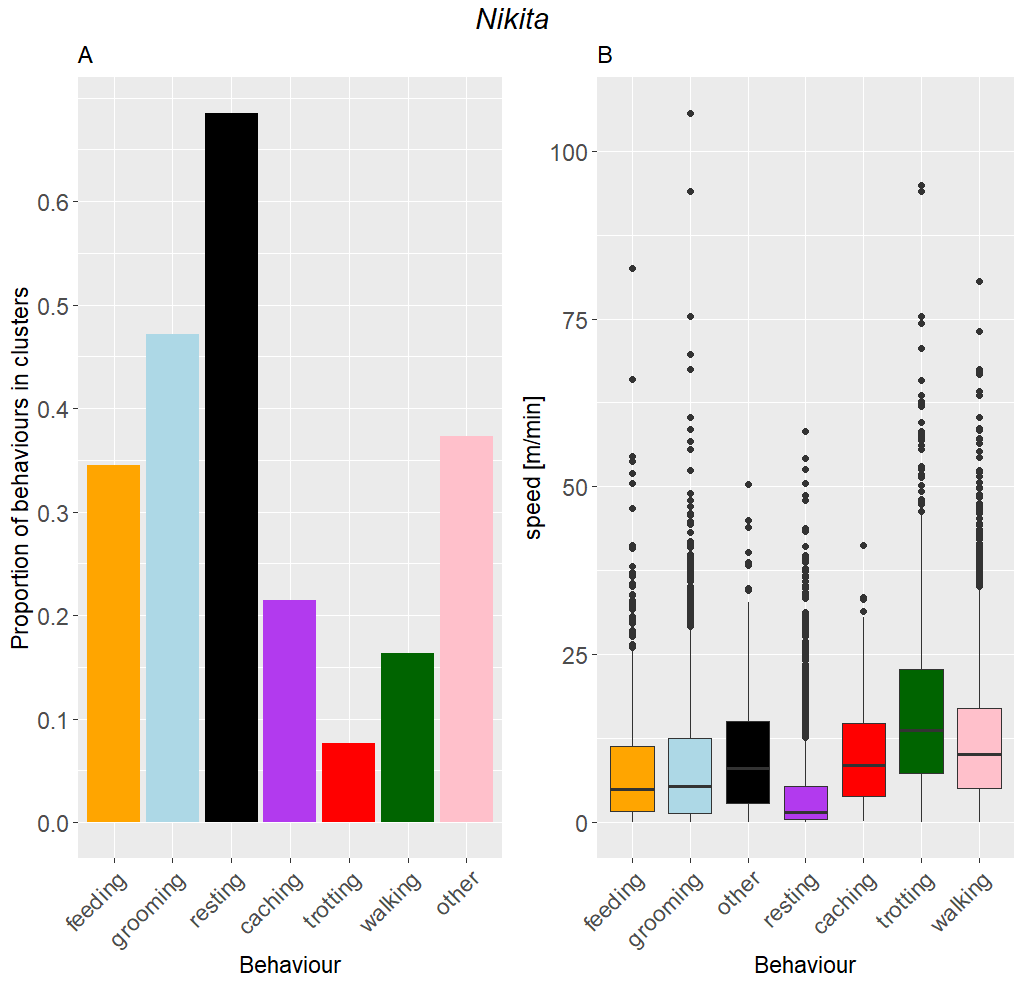

Supplement: S13 Fig — The numbers in the brackets indicate the sample size of each behaviour class. (A) Resting shows the highest association with GPS clusters (69%) and trotting the lowest (8%). (B) Behaviour prediction in relation to speed. Resting appears at significantly lower speeds than trotting (Wilcoxon rank sum test, W = 327626, p < 0.001). (TIF) [file pone.0227317.s013.tif]

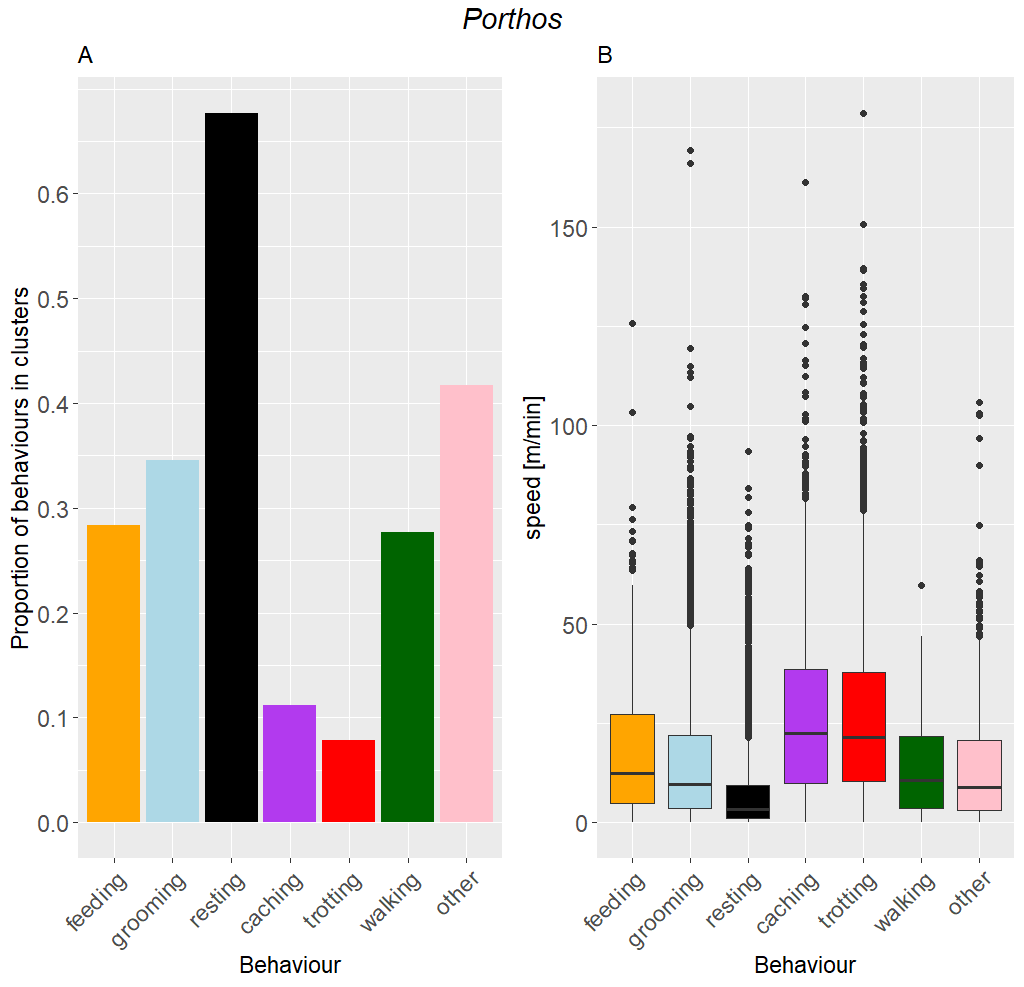

Supplement: S14 Fig — The numbers in the brackets indicate the sample size of each behaviour class. (A) Resting shows the highest association with GPS clusters (68%) and trotting the lowest (8%). (B) Behaviour prediction in relation to speed. Resting appears at significantly lower speeds than trotting (Wilcoxon rank sum test, W = 4454986, p < 0.001). (TIF) [file pone.0227317.s014.tif]

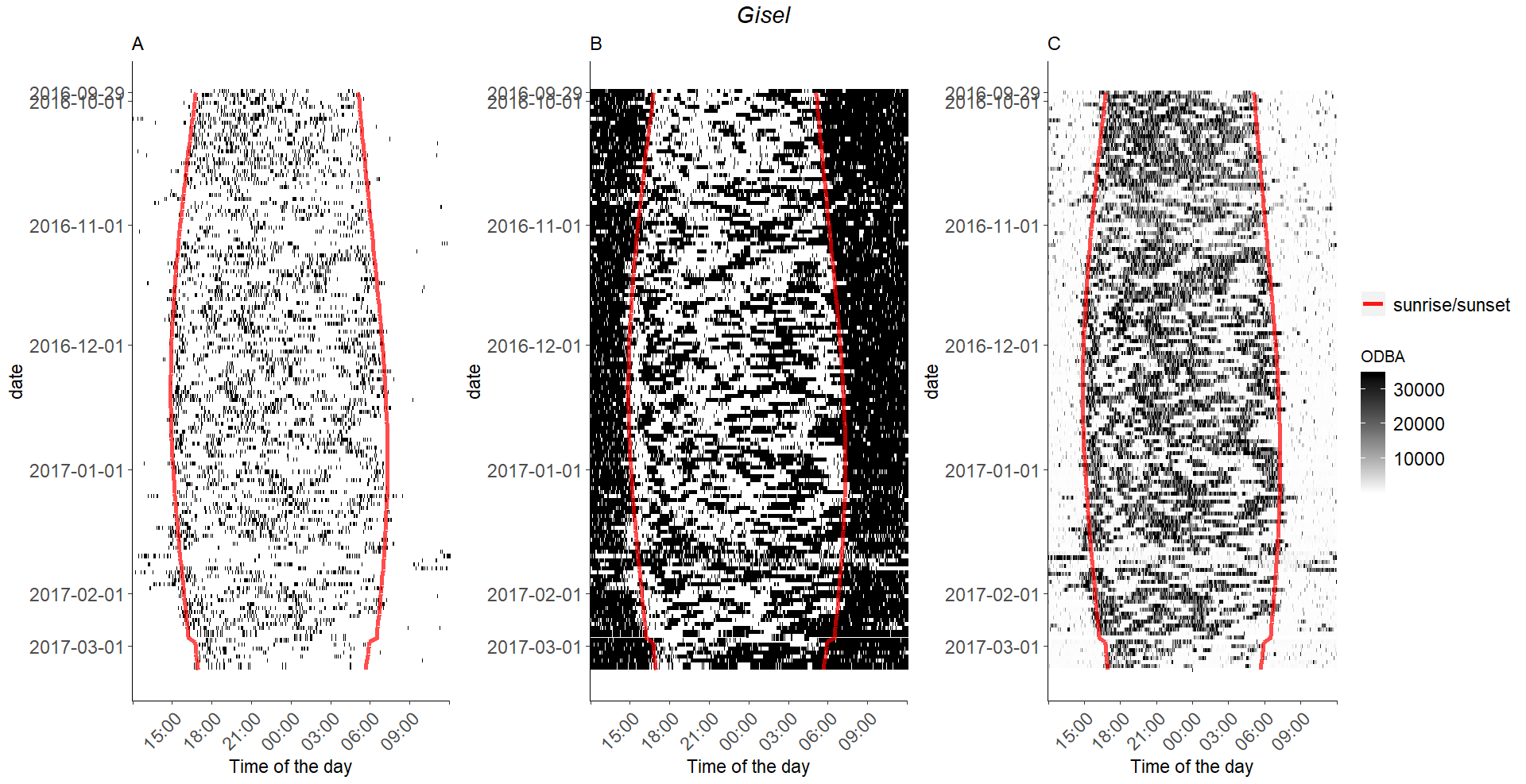

Supplement: S15 Fig — The red lines indicate the sunset and sunrise. (A) Black spaces indicate times at which trotting behaviour was predicted whereas white spaces indicate assignments of all other behaviours. Trotting is predominantly predicted during the night. (B) Black spaces indicate times at which resting behaviour was predicted whereas white spaces indicate assignments of all other behaviours. Resting is predominantly predicted during the day. The switch between trotting, resting and other behaviours is mostly oriented at the sunset and sunrise. (C) Higher ODBA values are indicated by darker spaces. ODBA values are higher at night than during daytime. Discontinuities in the red line are caused by missing data due to the logger not recording data at the time. (TIF) [file pone.0227317.s015.tif]

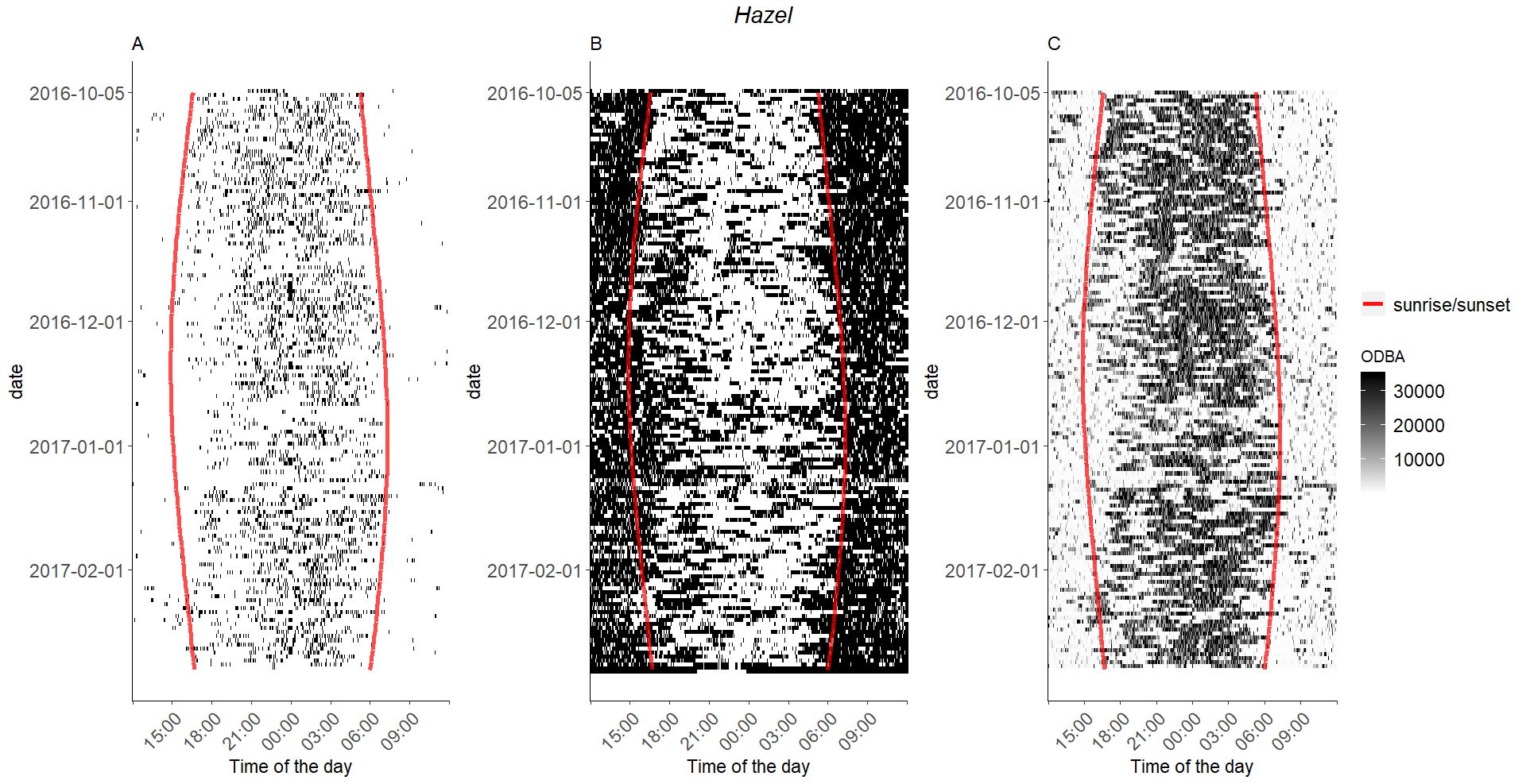

Supplement: S16 Fig — The red lines indicate the sunset and sunrise. (A) Black spaces indicate times at which trotting behaviour was predicted whereas white spaces indicate assignments of all other behaviours. Trotting is predominantly predicted during the night. (B) Black spaces indicate times at which resting behaviour was predicted whereas white spaces indicate assignments of all other behaviours. Resting is predominantly predicted during the day. The switch between trotting, resting and other behaviours is mostly oriented at the sunset and sunrise. (C) Higher ODBA values are indicated by darker spaces. ODBA values are higher at night than during daytime. (TIF) [file pone.0227317.s016.tif]

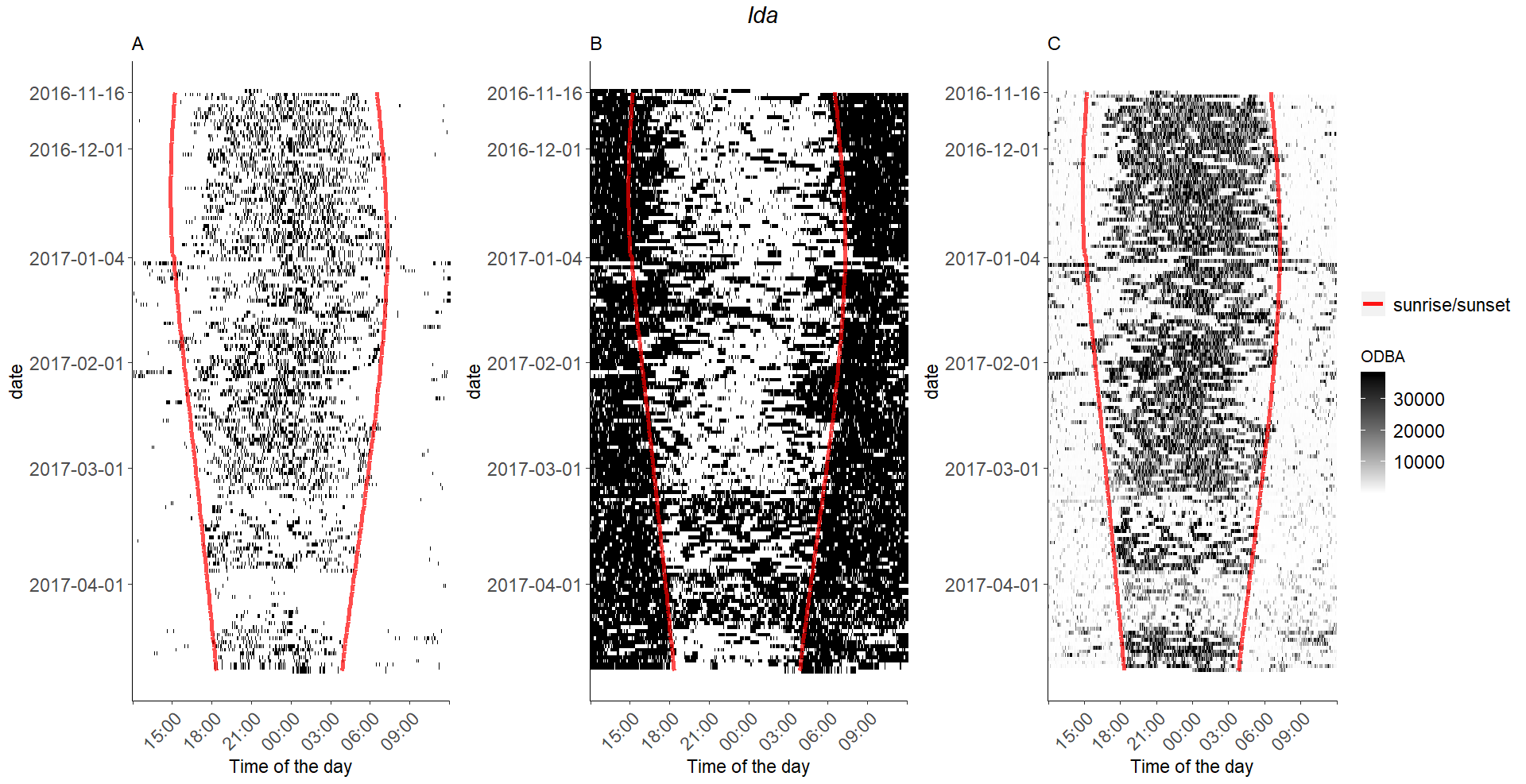

Supplement: S17 Fig — The red lines indicate the sunset and sunrise. (A) Black spaces indicate times at which trotting behaviour was predicted whereas white spaces indicate assignments of all other behaviours. Trotting is predominantly predicted during the night. (B) Black spaces indicate times at which resting behaviour was predicted whereas white spaces indicate assignments of all other behaviours. Resting is predominantly predicted during the day. The switch between trotting, resting and other behaviours is mostly oriented at the sunset and sunrise. (C) Higher ODBA values are indicated by darker spaces. ODBA values are higher at night than during daytime. (TIF) [file pone.0227317.s017.tif]

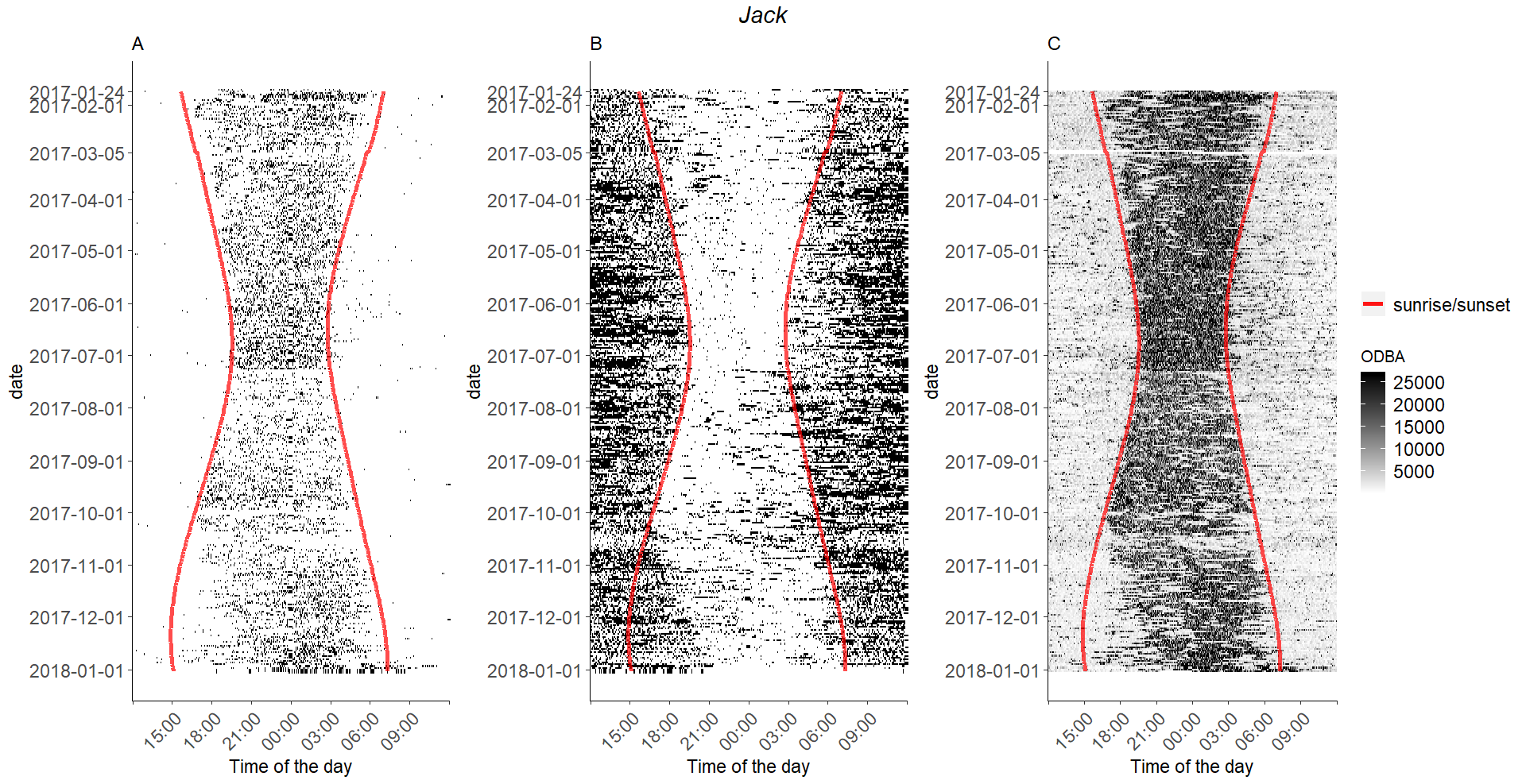

Supplement: S18 Fig — The red lines indicate the sunset and sunrise. (A) Black spaces indicate times at which trotting behaviour was predicted whereas white spaces indicate assignments of all other behaviours. Trotting is predominantly predicted during the night. (B) Black spaces indicate times at which resting behaviour was predicted whereas white spaces indicate assignments of all other behaviours. Resting is predominantly predicted during the day. The switch between trotting, resting and other behaviours is mostly oriented at the sunset and sunrise. (C) Higher ODBA values are indicated by darker spaces. ODBA values are higher at night than during daytime. (TIF) [file pone.0227317.s018.tif]

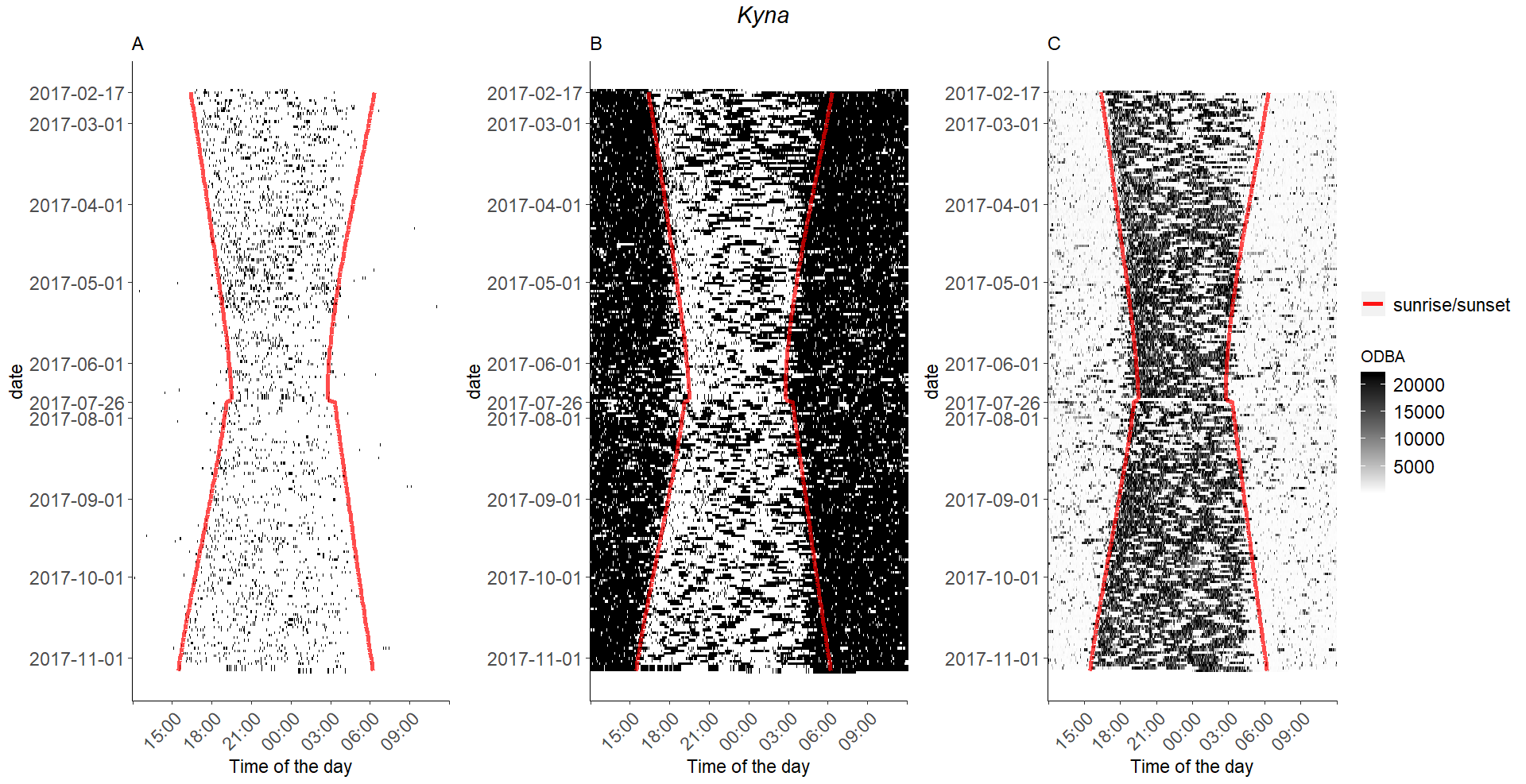

Supplement: S19 Fig — The red lines indicate the sunset and sunrise. (A) Black spaces indicate times at which trotting behaviour was predicted whereas white spaces indicate assignments of all other behaviours. Trotting is predominantly predicted during the night. (B) Black spaces indicate times at which resting behaviour was predicted whereas white spaces indicate assignments of all other behaviours. Resting is predominantly predicted during the day. The switch between trotting, resting and other behaviours is mostly oriented at the sunset and sunrise. (C) Higher ODBA values are indicated by darker spaces. ODBA values are higher at night than during daytime. Discontinuities in the red line are caused by missing data due to the logger not recording data at the time. (TIF) [file pone.0227317.s019.tif]

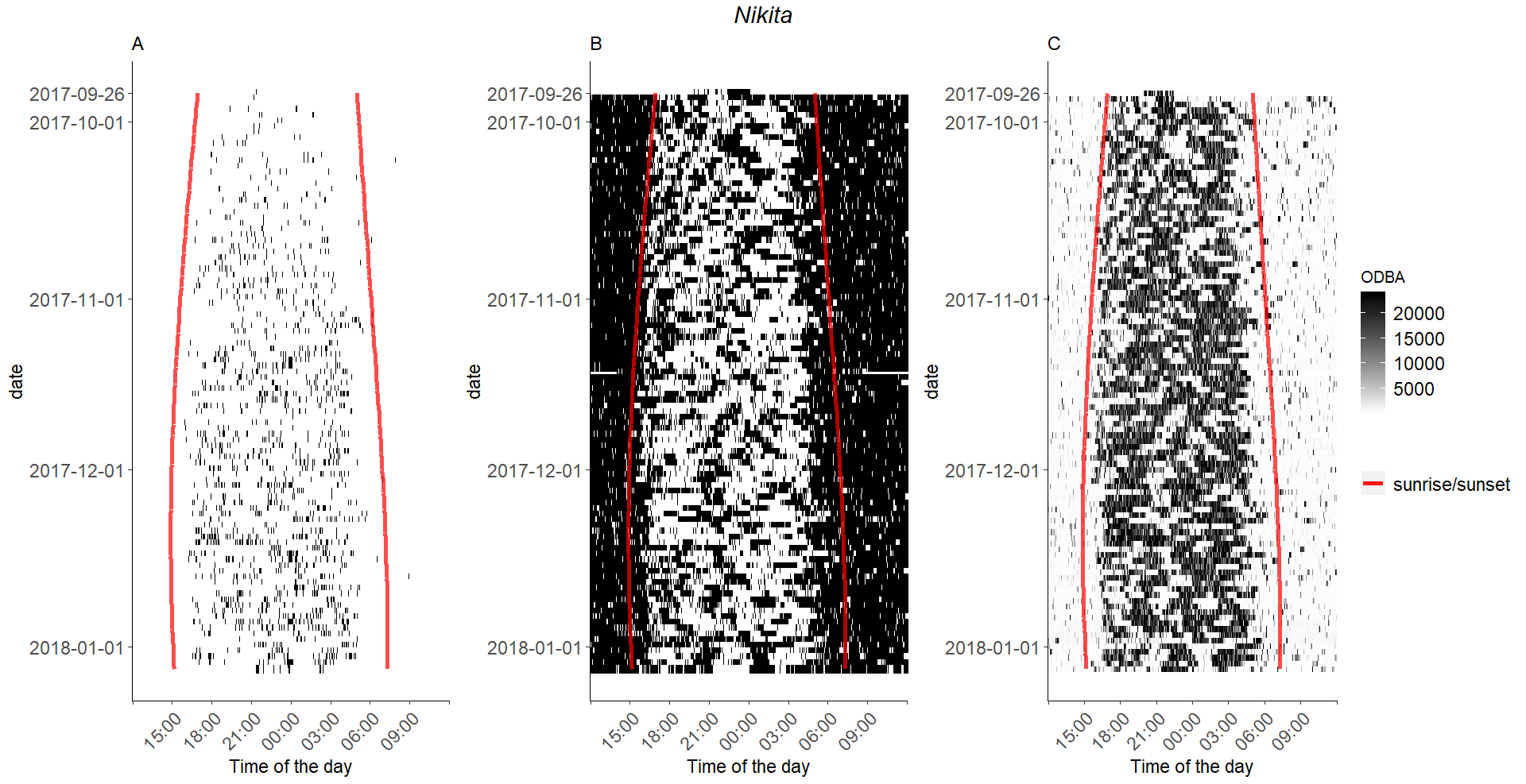

Supplement: S20 Fig — The red lines indicate the sunset and sunrise. (A) Black spaces indicate times at which trotting behaviour was predicted whereas white spaces indicate assignments of all other behaviours. Trotting is predominantly predicted during the night. (B) Black spaces indicate times at which resting behaviour was predicted whereas white spaces indicate assignments of all other behaviours. Resting is predominantly predicted during the day. The switch between trotting, resting and other behaviours is mostly oriented at the sunset and sunrise. (C) Higher ODBA values are indicated by darker spaces. ODBA values are higher at night than during daytime. (TIF) [file pone.0227317.s020.tif]

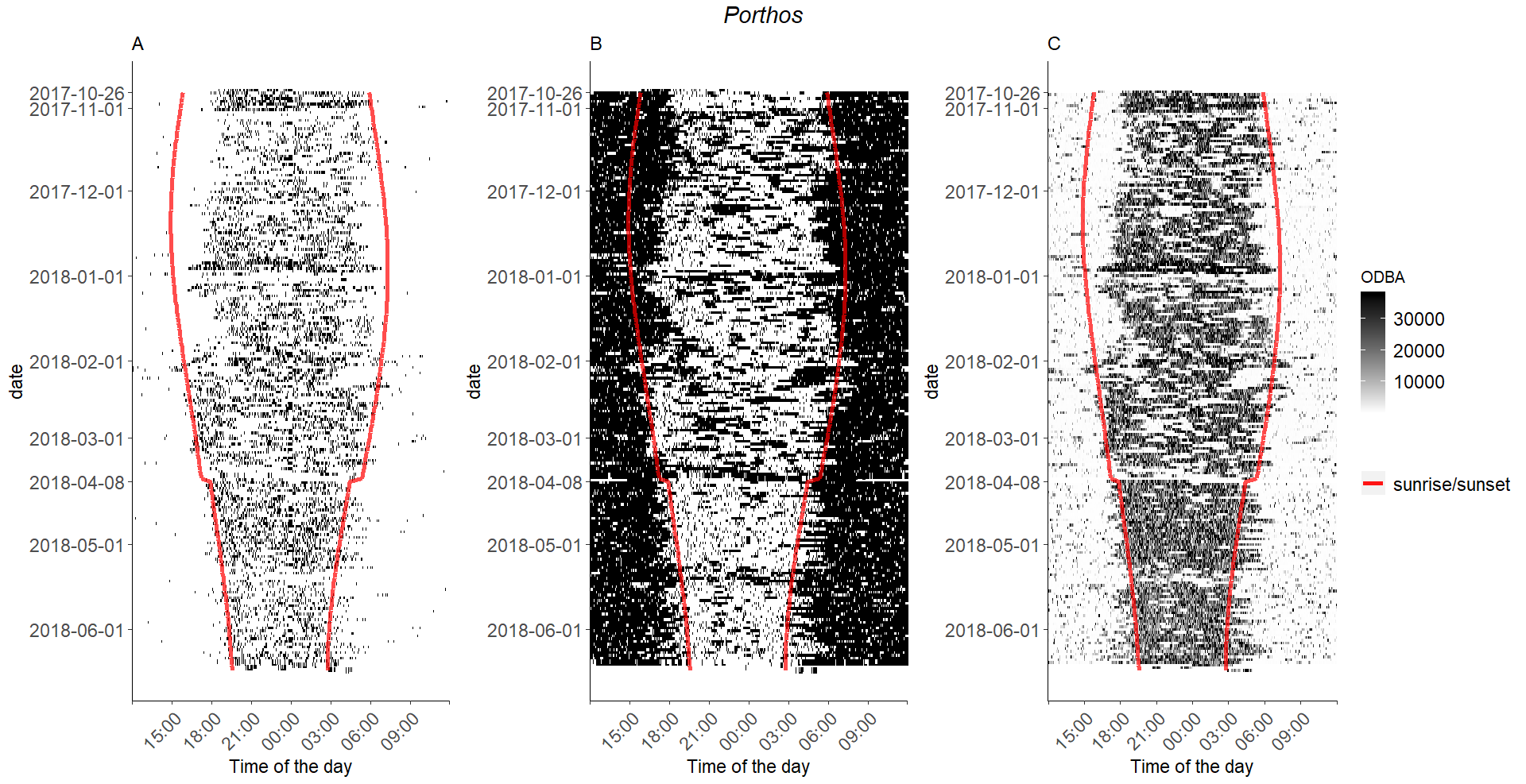

Supplement: S21 Fig — The red lines indicate the sunset and sunrise. (A) Black spaces indicate times at which trotting behaviour was predicted whereas white spaces indicate assignments of all other behaviours. Trotting is predominantly predicted during the night. (B) Black spaces indicate times at which resting behaviour was predicted whereas white spaces indicate assignments of all other behaviours. Resting is predominantly predicted during the day. The switch between trotting, resting and other behaviours is mostly oriented at the sunset and sunrise. (C) Higher ODBA values are indicated by darker spaces. ODBA values are higher at night than during daytime. Discontinuities in the red line are caused by missing data due to the logger not recording data at that time. (TIF) [file pone.0227317.s021.tif]
