# Supplementary material for: Machine learning goes wild: Using data from captive individuals to infer wildlife behaviours
Source: PLoS One. 2020 May 5;15(5):e0227317. doi: 10.1371/journal.pone.0227317 (PMC7200095; doi:10.1371/journal.pone.0227317)
Supplement: S1 Table — Behavioural observations were restricted to the listed categories. Each burst could only have one behaviour assigned. Observations not matching any description as well as observations of more than one behaviour per burst were excluded from the analysis. (DOCX) [file pone.0227317.s022.docx]

**S1 Table. Ethogram for the captive foxes – modified from** [**(Giese, 2016)**](https://www.zotero.org/google-docs/?NgRJYv)**.** Behavioural observations were restricted to the listed categories. Each burst could only have one behaviour assigned. Observations not matching any description as well as observations of more than one behaviour per burst were excluded from the analysis.

| **Behaviour** | **Description** |
| --- | --- |
| feeding | licking food items while standing on all four legs with the head held down;  chewing on food items while sitting on the hind legs with head held down or on level with shoulders;  chewing on food items while standing on all four legs with the head held down or head on level with shoulders |
| grooming | licking the body while lying on the stomach with head held up;  licking the body while sitting with the head held down;  scratching body parts with one hind leg while lying on the stomach with the head held up;  scratching body parts while sitting with the head held up or on level with shoulders;  nibbling body parts while lying on the stomach with head held up;  nibbling body parts while sitting on hind legs with head held up, on level with shoulders or held down;  nibbling body parts while standing on all four legs with the head held up or down |
| resting | lying on the stomach with the head resting on the ground;  lying on the stomach with the head held up, motionless or slightly moving from side to side;  sitting on hind legs with the head held up motionless or slightly moving from side to side;  standing on all four legs with the head held up motionless or slightly moving from side to side |
| caching | digging into the ground using one or both forelegs while standing on all four legs with the head held down  covering food items with soil and occasionally pressing it down using the snout while standing on all four legs with the head held down |
| trotting | moderately fast locomotion with all four feet losing contact with the ground for short moments with the head held up or on level with shoulders |
| walking | slow locomotion with at least three feet touching the ground at any given moment with the head on level with shoulders or held down and moving from side to side |
