# Supplementary material for: Machine learning goes wild: Using data from captive individuals to infer wildlife behaviours
Source: PLoS One. 2020 May 5;15(5):e0227317. doi: 10.1371/journal.pone.0227317 (PMC7200095; doi:10.1371/journal.pone.0227317)
Supplement: S2 Table — References refer to when the respective predictor was first introduced in the context of behaviour prediction. All predictors except the Fast Fourier Transformation can be considered summary statistics because they result in a single number. We add the complete Fast Fourier Spectrum as predictors. The total amount of predictors from the Fast Fourier Spectrum is therefore dependant on burst length. (DOCX) [file pone.0227317.s023.docx]

**S2 Table. List of all predictors and how they were calculated.** References refer to when the respective predictor was first introduced in the context of behaviour prediction. All predictors except the Fast Fourier Transformation can be considered summary statistics because they result in a single number. We add the complete Fast Fourier Spectrum as predictors. The total amount of predictors from the Fast Fourier Spectrum is therefore dependant on burst length.

| **Predictor** | **Calculation** | **Reference** |
| --- | --- | --- |
| Mean | mean of the x axis x  mean of the y axis y  mean of the z axis z |  |
| Standard deviation (sd) | sd of the x axis  sd of the y axis  sd of the z axis |  |
| Inverse coefficient of variation (ICV) | xsdofthexaxis  ysdoftheyaxis  zsdofthezaxis |  |
| Variation | 1n−1sd²ofthexaxis  1n−1sd²oftheyaxis  1n−1sd²ofthezaxis |  |
| Skewness | skewness() function in R, type = 3 of the x axis  skewness() function in R, type = 3 of the y axis  skewness() function in R, type = 3 of the z axis | [(Meyer et al., 2017)](https://www.zotero.org/google-docs/?0SMNmg) |
| Kurtosis | kurtosis() function in R, type = 3 of the x axis  kurtosis() function in R, type = 3 of the y axis  kurtosis() function in R, type = 3 of the z axis | [(Meyer et al., 2017)](https://www.zotero.org/google-docs/?ZtwIie) |
| q | x2+y2+z2 | [(Nathan et al., 2012)](https://www.zotero.org/google-docs/?eN2hdq) |
| Pitch | arctanyx2+z2180 | [(Collins et al., 2015)](https://www.zotero.org/google-docs/?iNsTmj) |
| Roll | arctanxy2+z2180 | [(Collins et al., 2015)](https://www.zotero.org/google-docs/?PjGFMF) |
| Overall body acceleration (ODBA) | 1nxn−x+1nyn−y+1nzn−z | [(Wilson et al., 2006)](https://www.zotero.org/google-docs/?sgMEtM) |
| Fast Fourier transformation (FFT) | fft() function in R of the x axis considering only the real numbers  fft() function in R of the y axis considering only the real numbers  fft() function in R of the z axis considering only the real numbers | [(R Core Team, 2018)](https://www.zotero.org/google-docs/?0jlCES) |
