# Supplementary material for: Machine learning goes wild: Using data from captive individuals to infer wildlife behaviours
Source: PLoS One. 2020 May 5;15(5):e0227317. doi: 10.1371/journal.pone.0227317 (PMC7200095; doi:10.1371/journal.pone.0227317)
Supplement: S4 Table — Columns show expected behaviours known from observation, rows show behaviours assigned by the RF. Values on the diagonal (bold) represent behaviours assigned correctly. All values off the diagonal are incorrect assignments that show which behaviours were confused with each other (e.g. 17 events of feeding were incorrectly classified as grooming). (DOCX) [file pone.0227317.s025.docx]

**S4 Table. Confusion matrix for the random forest (RF) validation.** Columns show expected behaviours known from observation, rows show behaviours assigned by the RF. Values on the diagonal (bold) represent behaviours assigned correctly. All values off the diagonal are incorrect assignments that show which behaviours were confused with each other (e.g. 17 events of feeding were incorrectly classified as grooming).

|  | **Expected** |  |  |  |  |  |
| --- | --- | --- | --- | --- | --- | --- |
| **Predicted** | **feeding** | **grooming** | **resting** | **caching** | **trotting** | **walking** |
| feeding | **77** | 10 | 1 | 13 | 0 | 7 |
| grooming | 20 | **319** | 47 | 2 | 2 | 9 |
| resting | 0 | 5 | **584** | 1 | 0 | 1 |
| caching | 12 | 6 | 1 | **40** | 0 | 9 |
| trotting | 1 | 0 | 0 | 2 | **52** | 2 |
| walking | 0 | 2 | 1 | 1 | 0 | **21** |
