# Supplementary material for: Machine learning goes wild: Using data from captive individuals to infer wildlife behaviours
Source: PLoS One. 2020 May 5;15(5):e0227317. doi: 10.1371/journal.pone.0227317 (PMC7200095; doi:10.1371/journal.pone.0227317)
Supplement: S5 Table — Columns show expected behaviours known from observation, rows show behaviours assigned by the ANN. Values on the diagonal (bold) represent behaviours assigned correctly. All values off the diagonal are incorrect assignments that show which behaviours were confused with each other (for example 15 events of resting were incorrectly predicted as feeding). (DOCX) [file pone.0227317.s026.docx]

**S5 Table. Confusion matrix for the artificial neural network (ANN) validation.** Columns show expected behaviours known from observation, rows show behaviours assigned by the ANN. Values on the diagonal (bold) represent behaviours assigned correctly. All values off the diagonal are incorrect assignments that show which behaviours were confused with each other (for example 15 events of resting were incorrectly predicted as feeding).

|  | **Expected** | | | | | |
| --- | --- | --- | --- | --- | --- | --- |
| **Predicted** | **feeding** | **grooming** | **resting** | **caching** | **trotting** | **walking** |
| feeding | **2934** | 252 | 19 | 199 | 0 | 86 |
| grooming | 198 | **9643** | 237 | 78 | 0 | 34 |
| resting | 15 | 282 | **19548** | 1 | 0 | 54 |
| caching | 103 | 62 | 32 | **1287** | 47 | 22 |
| trotting | 0 | 47 | 1 | 33 | **1656** | 88 |
| walking | 84 | 228 | 36 | 123 | 8 | **1157** |
