# Supplementary material for: Machine learning goes wild: Using data from captive individuals to infer wildlife behaviours
Source: PLoS One. 2020 May 5;15(5):e0227317. doi: 10.1371/journal.pone.0227317 (PMC7200095; doi:10.1371/journal.pone.0227317)
Supplement: S6 Table — Most of the times GPS and acceleration data were not recorded simultaneously. For the speed analysis we considered only acceleration data that was recorded within 10 seconds of a GPS recording. (DOCX) [file pone.0227317.s027.docx]

**S6 Table. Proportion of data used for the speed analysis.** Most of the times GPS and acceleration data were not recorded simultaneously. For the speed analysis we considered only acceleration data that was recorded within 10 seconds of a GPS recording.

| **Individual** | **Proportion of data used** |
| --- | --- |
| Porthos | 0.15 |
| Kyna | 0.08 |
| Nikita | 0.15 |
| Gerlinde | 0.05 |
| Gisel | 0.11 |
| Hazel | 0.10 |
| Ida | 0.11 |
| Jack | 0.09 |
| Que | 0.07 |
